# What drives polymer adsorption into MOF nanopores? Insights into the role of solvents from titration calorimetry

**DOI:** 10.1039/d6sc03917k

**Published:** 2026-10-19

**Authors:** Yu Kono, Takashi Uemura, Nobuhiko Hosono

**Affiliations:** a Department of Applied Chemistry, Graduate School of Engineering, The University of Tokyo 7-3-1 Hongo, Bunkyo-ku Tokyo 113-8656 Japan uemurat@g.ecc.u-tokyo.ac.jp nhosono@g.ecc.u-tokyo.ac.jp

## Abstract

Spontaneous infiltration of polymer chains into nanometer-sized pores of metal–organic frameworks (MOFs) has emerged as an intriguing phenomenon with potential applications in polymer recognition, separation, and nanocomposite fabrication. Despite the substantial conformational entropy loss expected upon confinement within sub-nanometer channels, polymers are known to penetrate MOF nanopores and adsorb from solution. However, the thermodynamic origin of this counterintuitive process remains unresolved, largely due to the difficulty of disentangling the interplay of interactions among the polymer, framework, and solvent. Here, we study the thermodynamics governing polymer adsorption into MOF nanopores using isothermal titration calorimetry (ITC) in a series of organic solvents. Estimation of apparent Δ*G*, Δ*H*, and Δ*S* from ITC analysis using simple Langmuir-type fitting models indicates that, although adsorption is exothermic, the overall free energy change is accompanied by a favorable entropic contribution. Notably, the apparent entropy gain is largely independent of solvent identity, whereas the enthalpic term varies significantly with solvent. These findings suggest that solvent release from the nanopores and polymer desolvation plausibly contribute to the adsorption driving force, with their relative importance governed by solvent choice rather than polymer–framework interactions alone. This result overturns the conventional enthalpy-driven interpretation. Analysis of the individual titration profiles further provides kinetic insight, supporting a two-step adsorption mechanism consisting of initial surface association followed by intrapore diffusion. This study establishes a solvent-mediated thermodynamic framework for understanding polymer adsorption in nanoporous materials and offers guiding principles for the rational control of polymer infiltration in confined environments.

## Abstract 2

Calorimetric analysis reveals the central role of solvents in polymer adsorption into MOF nanopores. The favorable entropy gain may reflect solvent release and polymer desolvation, while solvent identity strongly affects the enthalpy and kinetics.

## Graphical abstract

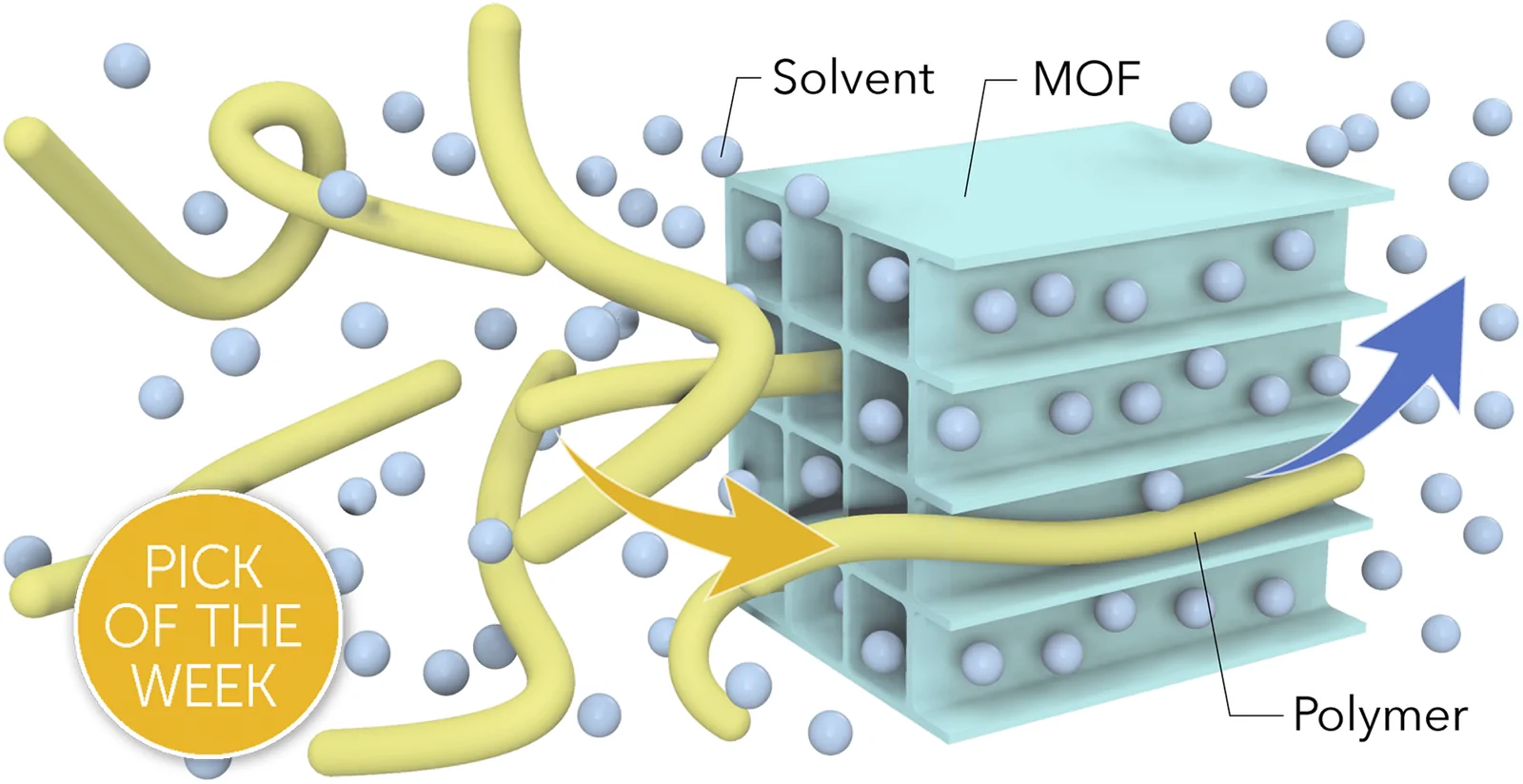

## Introduction

Molecular adsorption in porous materials underpins a wide range of applications, including storage, purification, and separation, as well as nanocomposite fabrication. The spectrum of adsorbates spans from small gas molecules to large macromolecules, reflecting the diverse functional demands across scientific and industrial fields. Among porous materials, metal–organic frameworks (MOFs) have attracted particular attention owing to their exceptionally high surface areas and precisely tunable pore environments.^1^ In recent years, the adsorption of polymers into nanometer-sized MOF pores has emerged as an intriguing and technologically significant phenomenon.^2,3^ Remarkably, even long-chain polymers have been reported to spontaneously infiltrate MOF nanopores from solution.^4^ This behavior has enabled new strategies not only for organic/inorganic nanocomposite fabrications^5–7^ but also high-precision polymer separation techniques, in which subtle structural features of polymer chains are discriminated through nanoscale threading events within the pores.^8–14^

Despite rapid progress and expanding practical applications, the thermodynamic driving force underlying the spontaneous infiltration of polymer chains into such confined nanopores remains poorly understood. A wide range of polymers, including synthetic polymers such as polyethylene glycol (PEG),^4,15–17^ poly(4-vinylpyridine),^10^ poly(methyl methacrylate),^14,18^ and poly(3-hexylthiophene),^13^ as well as biopolymers such as proteins^11,19,20^ and DNA/RNA,^21–23^ have been shown to penetrate MOF pores whose apertures are smaller than the hydrodynamic dimensions of the polymer chains in solution. During infiltration, polymer chains thread through pore apertures comparable to their thickness, typically entering from the chain termini, followed by diffusion into the interior channels of the MOF crystals where they become confined.^24^ Notably, strong solvent dependence has frequently been observed in these systems,^4,14,17^ suggesting that solvation effects play a critical yet unresolved role in governing the adsorption process.

From a polymer physics perspective, confinement within sub-nanometer channels imposes severe conformational constraints, forcing partial chain elongation and reducing accessible configurations.^25^ Such confinement should result in a substantial loss of conformational entropy. Accordingly, the polymer infiltration process, especially under solvent-free conditions, has generally been interpreted as being predominantly enthalpy-driven, where favorable polymer–MOF interactions are assumed to compensate for the large entropic penalty.^24,26^ For example, molten PEG has been shown to spontaneously infiltrate sub-nanoporous MOF crystals under solvent-free conditions at elevated temperatures upon simple mixing with the MOF crystals. Such systems typically exhibit exothermic heat evolution upon infiltration, supporting an enthalpy-driven mechanism.^8,24,26,27^

In contrast, the thermodynamic basis of polymer adsorption in the solution phase has remained largely unexplored due to the complexity arising from strong solvent dependence, which limits a fundamental understanding of what truly drives polymer adsorption in MOF nanopores.^2–4^ This knowledge gap arises largely from experimental challenges in directly quantifying entropic contributions and in disentangling the complex interplay among the three key components of the system: polymer, MOF, and solvent molecules (Fig. 1).

**Fig. 1:**
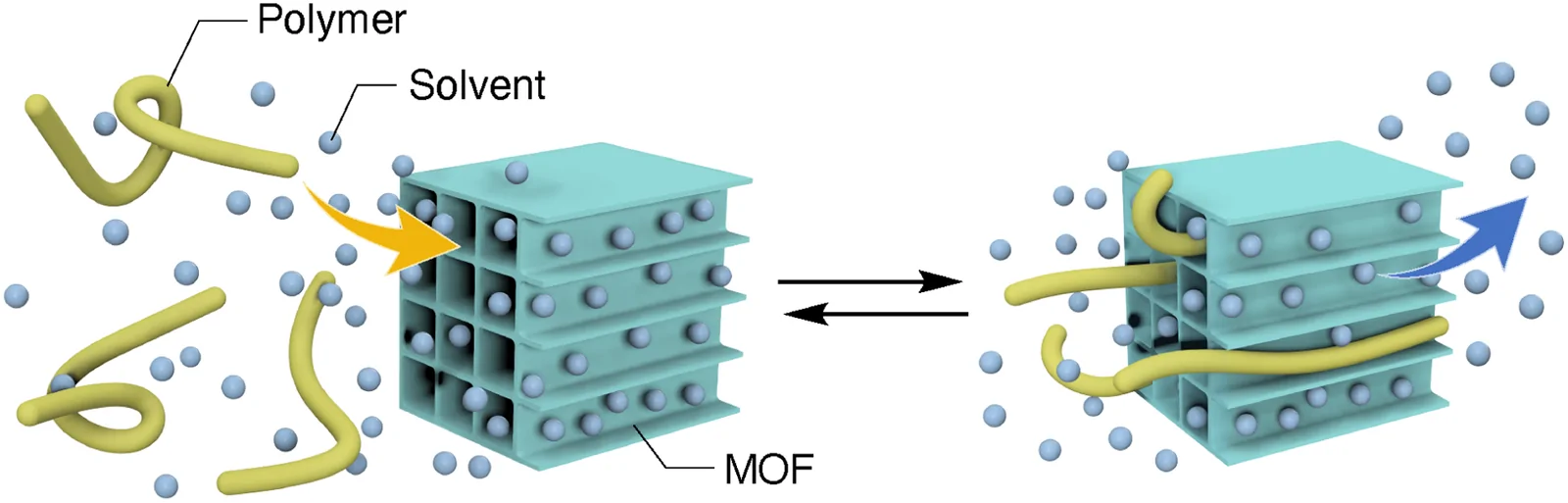
Schematic illustration of polymer adsorption into MOF nanopores from solution, highlighting the interplay among MOF–polymer, MOF–solvent, and polymer–solvent interactions governing the polymer infiltration event.

In this study, we investigate the thermodynamic origin of polymer adsorption into MOF nanopores in solution using isothermal titration calorimetry (ITC). ITC is a powerful technique for probing thermodynamics in supramolecular systems, as it directly measures the heat exchanged during association processes. From calorimetric data, equilibrium constants and the corresponding thermodynamic parameters, *i.e.* enthalpy (Δ*H*), entropy (Δ*S*), and Gibbs free energy (Δ*G*), can be simultaneously obtained by fitting to an association model,^28^ along with the basic thermodynamic relationship:Δ*G* = Δ*H* − *T*Δ*S* = −*RT* ln *K*

While ITC has been widely applied to investigate molecular interactions and host–guest chemistry,^29–35^ its application to solid adsorption systems remains limited,^16,36–44^ and predominantly restricted to aqueous media. By applying ITC across different organic solvents, we access previously elusive entropic contributions and investigate the solvent-dependent thermodynamic landscape of polymer adsorption. Furthermore, time evolution of the ITC thermograms provides kinetic insight, supporting a two-step mechanism proposed in earlier studies.^4,14^ In this mechanism, polymer chains initially adsorb onto the external surface of the MOF crystals and subsequently diffuse into the nanopores to occupy the internal channels. This sequential process is considered as a crucial step underlying discriminative polymer adsorption in the separation applications.^4^

As a model system, we selected a sub-nanoporous MOF [Zn_2_(ndc)_2_(ted)]_*n*_ (hereafter denoted as 1; ndc = 1,4-naphthalenedicarboxylate; ted = triethylenediamine)^45^ as the host and PEG as the guest polymer (Fig. 2). MOF 1 features a pillared-layer structure with pseudo-one-dimensional channels along the *c*-axis and main pore apertures of ∼0.57 nm.^45^ Previous studies have demonstrated pronounced solvent-dependent adsorption behavior of PEG in 1: adsorption proceeds readily in ethanol (EtOH), 1-propanol (PrOH), 1-butanol (BuOH) and acetonitrile (MeCN), whereas significantly reduced adsorption is observed in toluene (Fig. S1).^4^

**Fig. 2:**
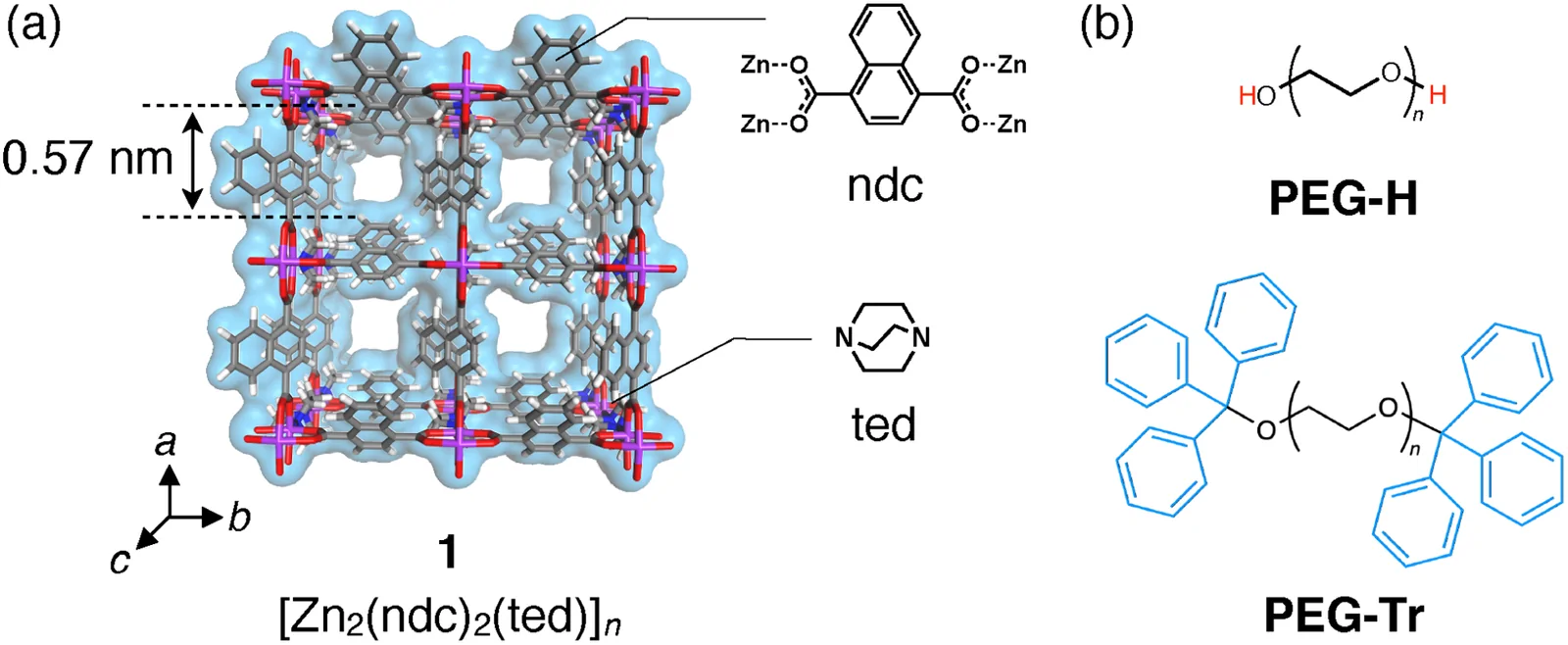
(a) Structure of [Zn_2_(ndc)_2_(ted)]_*n*_ (1), showing the main pore aperture of 0.57 nm. Color code: C, gray; N, blue; O, red; H, white; Zn, purple. (b) Chemical structures of hydroxy-terminated PEG, PEG-H, and trityl-terminated PEG, PEG-Tr.

Through systematic ITC measurements in these solvents and investigation on the fitting models, we obtained the thermodynamic parameters associated with PEG adsorption into 1 from Langmuir-type models. Significant heat evolution was observed in alcohols and MeCN, yielding negative Δ*G* values and confirming the spontaneous nature of the process in these media. Although adsorption was generally exothermic in all examined cases (negative Δ*H*), the entropic contribution (*T*Δ*S*) was also found to contribute favorably to the free energy change. This observation contrasts with the conventional view that molecular adsorption in organic media is primarily enthalpy-driven.^4^ In aqueous systems, molecular adsorption is often entropy-driven due to the desolvation effect, where structured water molecules are released upon adsorption, leading to a large entropy increase. Notably, in the present system, even in non-hydrogen-bond-donating MeCN, polymer adsorption in MOF nanopores follows an entropically-favored process.

This unexpected entropy gain may arise, at least in part, from the release of pore-confined and PEG-associated solvent molecules upon polymer inclusion (Fig. 1). While Δ*H* varies significantly with solvent identity, the *T*Δ*S* term remains favorable and nearly constant across solvents that permit adsorption. These results are consistent with possible contributions from solvent displacement from the MOF nanopores and PEG desolvation to the favorable entropy change, which itself shows relatively little dependence on solvent identity.

These findings provide important design guidelines for achieving polymer infiltration into extremely narrow pores. They highlight that solvent choice is a key factor, as solvent molecules, although often overlooked, play an important role in determining the overall thermodynamics of the adsorption process.

## Results and discussion

### ITC measurements of PEG infiltration into 1 from solution

The heat associated with PEG adsorption into 1 was directly measured by ITC in a series of organic solvents, namely EtOH, PrOH, BuOH, MeCN, and toluene. 1 was synthesized *via* a conventional solvothermal method,^45^ affording microcrystalline particles with an average size of 19.7 ± 7.2 µm (Fig. S2, SI Section 2). Scanning electron microscopy (SEM) revealed a cuboid crystal morphology, and N_2_ adsorption analysis at 77 K confirmed permanent porosity consistent with previously reported values (Fig. S3 and S4).^45^ Powder X-ray diffraction (PXRD) measurements verified that the crystalline structure of 1 remained intact in all solvents used for the ITC experiments, demonstrating its chemical stability under the measurement conditions (Fig. S5).

Hydroxy-terminated PEG with a molecular weight (MW) of 2 kg mol^−1^ (Table S1, hereafter denoted as PEG-H) was employed as the representative guest polymer. PEG was selected because its adsorption into microporous materials, including MOFs^4,15–17^ and zeolites,^16,46^ has been extensively studied, allowing direct comparison with prior reports.^4^

For the initial ITC analysis, EtOH was selected as the solvent because it exhibited the highest adsorption capacity in previous isotherm measurements (Fig. S1).^4^ In a typical experiment, a defined volume of PEG solution in EtOH (*C*_0_ = 12 mg mL^−1^) was successively injected into an EtOH suspension of 1 at 313 K, and the resulting heat flow was recorded (see SI Section 3 for the detailed measurement conditions). The raw thermogram displayed exothermic peaks upon each injection, reflecting the adsorption of PEG-H into 1 from solution (Fig. 3). Notably, two distinct features were observed in the titration thermogram for PEG-H, namely a sharp initial peak immediately after the injection, followed by a broader and more gradual heat evolution persisting for over 80 min (Fig. 3c and f).

**Fig. 3:**
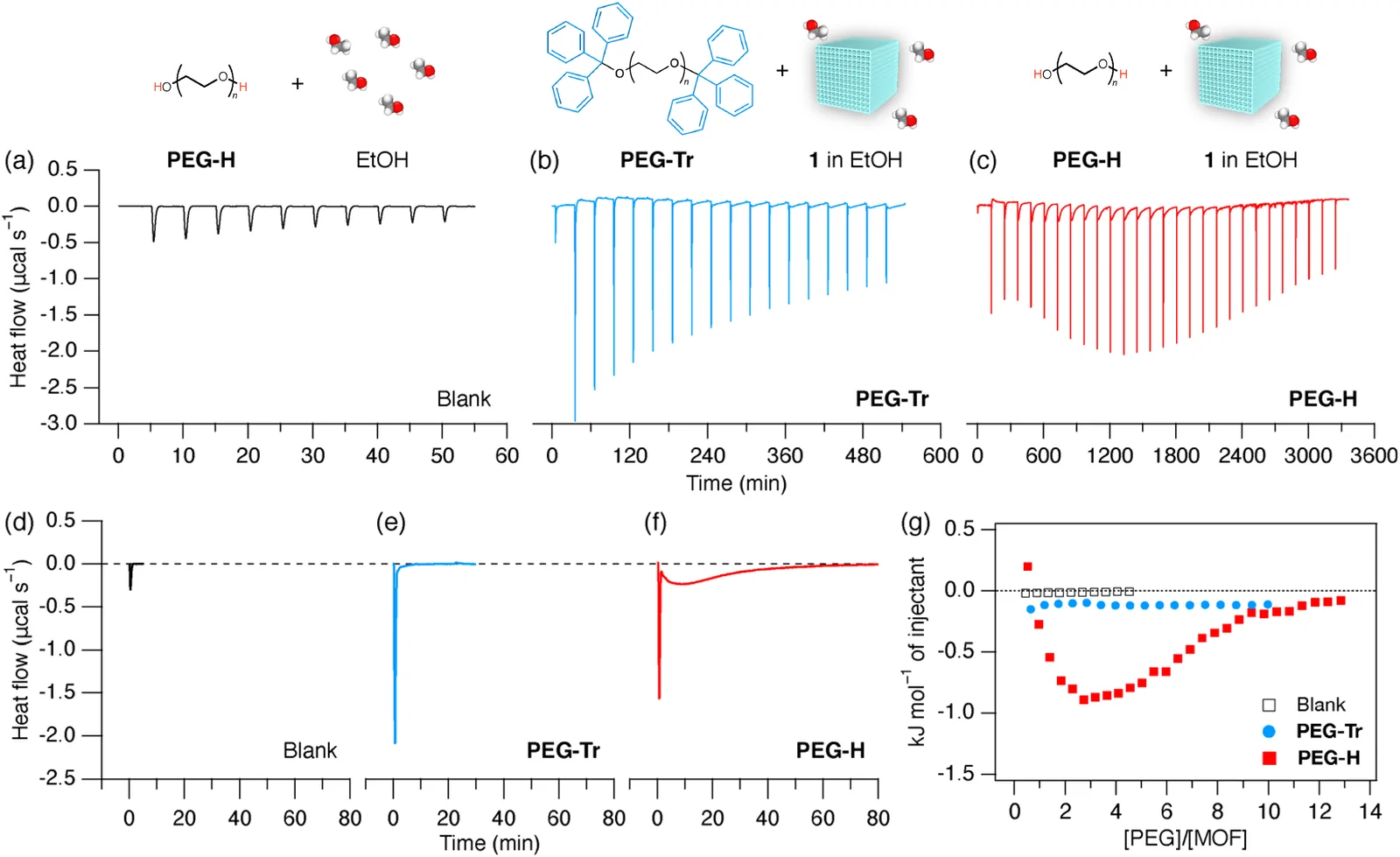
(a–c) Representative ITC thermograms at 313 K for (a) EtOH injected with an EtOH solution of PEG-H (blank experiment), and for EtOH suspensions of 1 injected with EtOH solutions of (b) PEG-Tr and (c) PEG-H. (d–f) Enlarged thermograms showing the fifth injection peak for (d) the blank, (e) PEG-Tr, and (f) PEG-H experiments at 313 K. (g) Normalized injection heat profiles as a function of the molar ratio ([PEG]/[MOF]) for the blank (open squares), PEG-Tr (aqua-blue circles), and PEG-H (red squares) at 313 K, obtained by integration of the ITC thermograms. The concentrations of MOF and PEG, denoted as [MOF] and [PEG], are defined as the molar amounts of MOF unit cells and PEG repeating units (–CH_2_CH_2_O–), respectively.

To verify that the observed heat originated from PEG infiltration rather than dilution effects, control experiments were conducted in the absence of MOF particles. The blank thermograms showed only sharp and small exothermic signals corresponding to the heat of dilution of PEG (Fig. 3a and d), confirming that the dominant and broad signals in the presence of 1 arise from PEG–MOF interactions. Integration of each thermal signal further confirmed that the dilution heat was negligible under EtOH conditions, approximately 0.01–0.02 kJ mol^−1^ (Fig. 3g).

To further substantiate PEG infiltration into the nanopores of 1, ITC measurements were performed using trityl-terminated PEG (PEG-Tr) instead of PEG-H (Fig. 2 and S6). The bulky trityl end group, approximately 1 nm in size, exceeds the pore aperture of 1 and is therefore expected to inhibit intrapore infiltration while still permitting surface adsorption.^8,27^ For PEG-Tr, the thermograms showed a small heat evolution persisting within a few minutes in addition to a sharp initial dilution heat (Fig. 3b and e), resulting in a small and nearly constant exothermic signal of approximately 0.1 kJ mol^−1^ (Fig. 3g). This exotherm was observed irrespective of injection cycle, which is attributed to adsorption on the external surface of the MOF crystals.^8,27^ In contrast, PEG-H exhibited significantly larger heat evolution of approximately 1 kJ mol^−1^, which gradually converged to a value comparable to that of PEG-Tr at higher PEG loadings (Fig. 3g).

These ITC results strongly support the two-step adsorption mechanism proposed in previous studies, consisting of (1) rapid surface adsorption and (2) slower infiltration into the internal nanopores, with the latter serving as the rate-determining step.^4,14^ The slow diffusion process is consistent with the narrow sub-nanometer pore aperture of 1.^46^ Furthermore, the relatively small heat associated with the surface adsorption indicates that substantial energetic stabilization is achieved primarily upon infiltration and confinement of PEG chains within the internal pores of MOF crystals.

A further notable feature under EtOH conditions is the dependence of heat on PEG loading. The integrated heat profile shown in Fig. 3g exhibits a concave shape rather than the sigmoidal behavior typically observed for simple host–guest binding systems.^28^ Intriguingly, the first injection produces an endothermic signal, whereas subsequent injections become exothermic (Fig. 3g and S7). This trend suggests that a favorable entropic contribution is important especially during the initial stage, while later adsorption steps gain increasing enthalpic stabilization. Such behavior could suggest a cooperative adsorption mechanism in which pre-adsorbed polymer chains modify the pore environment, thereby facilitating subsequent infiltration events. Notably, a similar adsorption profile has been reported for PEG infiltration into a zeolite (silicalite-1) with a comparable pore size of 0.55 nm in aqueous media.^16^ Possible origins of this cooperativity include partial solvent displacement from the solvated chains during the first infiltration step and favorable polymer–polymer interactions within the confined channels, both of which may lower the energetic barrier for further adsorption.

However, the presence of this cooperative effect in the present system remains tentative. The concave heat profile was observed only in EtOH, and was not evident in the other solvents examined, namely PrOH, BuOH, and MeCN (Fig. S8) (*vide infra*). This solvent dependence indicates that additional thermodynamic contributions must be considered. In particular, the overall enthalpy change may also include contributions from desolvation of PEG chains upon insertion, desorption of solvent molecules released from the MOF pores, and possible hydrogen-bonding reformation of the solvent molecules released, in addition to PEG–MOF and PEG–PEG interactions.

### Fitting model investigation and stoichiometry

The ITC measurements performed in PrOH, BuOH, MeCN, and toluene showed a clear correspondence between the reported adsorption isotherms (Fig. S1)^4^ and the calorimetric responses. In toluene, no detectable heat transfer other than the dilution heat was observed (Fig. S9), consistent with previous findings that PEG adsorption into 1 hardly occurs in this solvent.^4^ In contrast, significant heat evolution, particularly exothermic signals, was detected in PrOH, BuOH, and MeCN, where PEG adsorption proceeds similarly to that observed in EtOH (Fig. 4). Under these solvent conditions, the integrated heat profiles exhibited a characteristic sigmoidal profile, typical of host–guest binding systems (Fig. S8).^28^

**Fig. 4:**
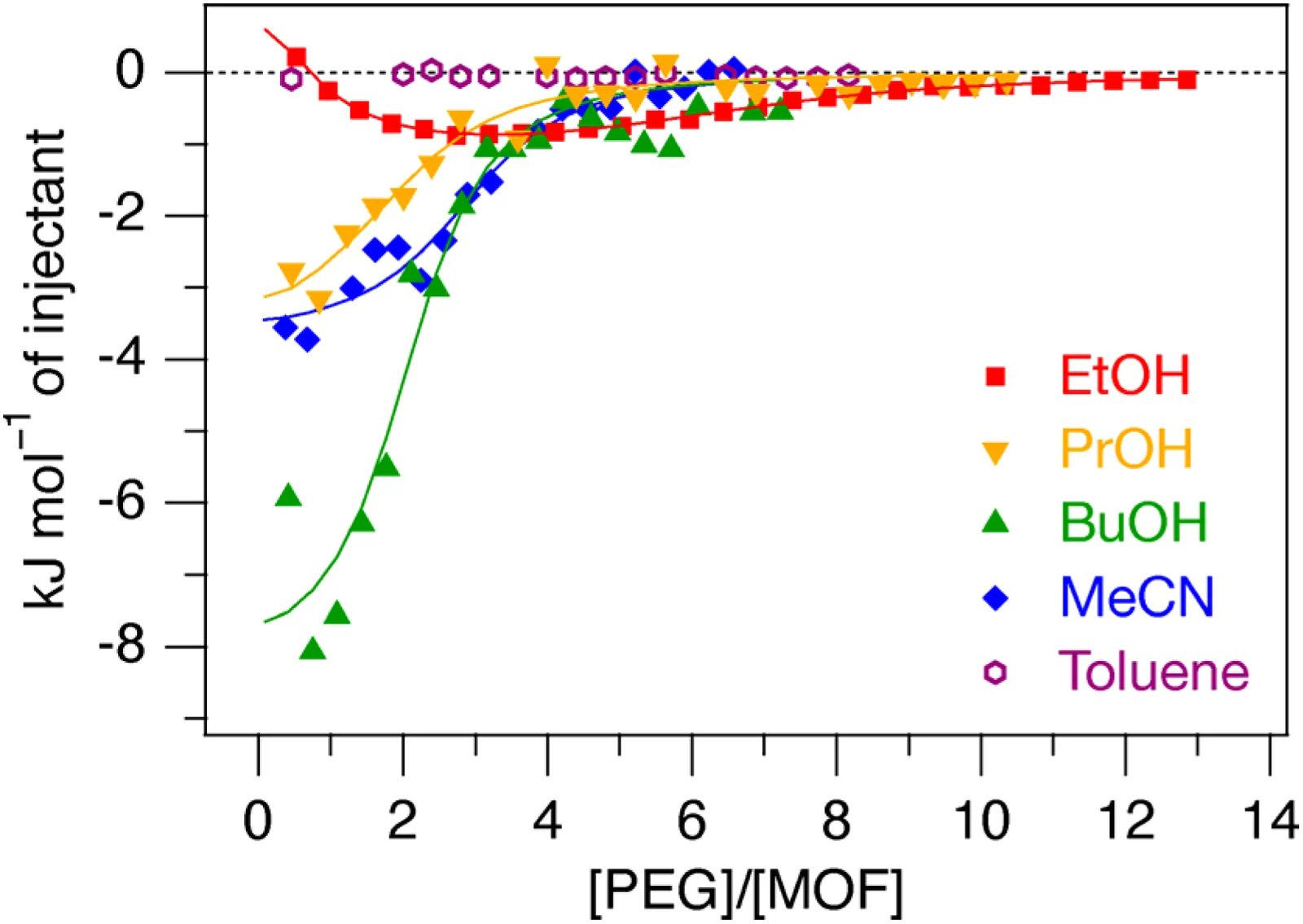
Normalized injection heat profiles as a function of the molar ratio for PEG-H adsorption into 1 at 313 K, measured in different solvents: EtOH (red square), PrOH (orange down triangle), BuOH (green up triangle), MeCN (blue diamond), and toluene (purple hexagon). Solid lines represent fits to the two-set model for EtOH and the one-set model for PrOH, BuOH, and MeCN.

The experimentally obtained heat profiles for EtOH, PrOH, BuOH, and MeCN were subsequently analyzed using several fitting models to examine which model describes the experimental results well. At first, a simple Langmuir-type model which determines a single set of three independent parameters: the apparent association constant *K*, Δ*H*, and stoichiometry *N* (namely, hereafter referred to as the one-set model), and a two-set model assuming two sets of the three parameters for two independent adsorption sites (Mode 1 and 2, *vide infra*) were considered.^28^ In the model analysis, adsorption was treated as an association event between a MOF unit cell and a PEG repeating unit. The data for PrOH, BuOH, and MeCN were well fitted by the theoretical sigmoidal curves using the one-set model (Fig. 4 and Tables 1, 2, S2, S3), whereas the two-set model failed to converge to physically meaningful fitting parameters. In contrast, the data for EtOH, which exhibited complex heat evolution during the early stage of titration, was well described by the two-set model (Fig. 4, S7 and Tables 1, 2), whereas the one-set model failed to reproduce the experimental profile.

**Table 1:** Stoichiometric values and equilibrium adsorption amounts of PEG-H to 1 in different solvents at 313 K, compared with estimated values from the reported isotherm

Solvent	*N* a ([PEG]/[MOF])	*q* b (g g^−1^)	*q*_L_ c (g g^−1^)
EtOH (Mode 1)^d^	0.41 ± 0.32	0.03	—
EtOH (Mode 2)^d^	5.96 ± 0.19	0.39	—
EtOH-total (Mode 1 & 2)	6.37	0.42	0.28
PrOH^e^	1.92 ± 0.21	0.13	0.16
BuOH^e^	2.06 ± 0.15	0.13	0.15
MeCN^e^	2.91 ± 0.14	0.19	0.20

a Determined through ITC measurements and fitting analysis. Errors represent the fitting uncertainties obtained from the nonlinear least-squares fit.

b PEG adsorption amount corresponding to *N*.

c Determined based on the reported isotherm at the corresponding PEG concentrations of the adsorption end point.^4^

d Obtained from the two-set model.

e Obtained from the one-set model.

**Table 2:** *K* and thermodynamic parameters obtained from the fitting analysis for PEG-H adsorption into 1

Solvent	Temperature (K)	*K* a (10^3^ M^−1^)	Δ*G*^b^ (kJ mol^−1^)	Δ*H*^a^ (kJ mol^−1^)	*T*Δ*S*^b^ (kJ mol^−1^)
EtOH (Mode 1)^c^	313	2.66 ± 1.42	−20.5 ± 1.4	4.79 ± 5.82	25.3 ± 4.5
EtOH (Mode 2)^c^	313	0.35 ± 0.04	−15.3 ± 0.3	−1.38 ± 0.16	13.9 ± 0.4
PrOH^d^	313	1.58 ± 0.69	−19.2 ± 1.1	−3.79 ± 0.55	15.4 ± 1.6
BuOH^d^	313	3.55 ± 1.73	−21.3 ± 1.3	−8.30 ± 0.82	13.0 ± 2.0
MeCN^d^	313	1.26 ± 0.43	−18.6 ± 0.9	−3.68 ± 0.23	14.9 ± 1.1
MeCN^d^	308	2.74 ± 0.50	−20.3 ± 0.5	−4.12 ± 0.10	16.2 ± 0.5
MeCN^d^	303	29.0 ± 19.3	−25.9 ± 1.7	−4.34 ± 0.14	21.6 ± 1.7

a Errors represent the fitting uncertainties obtained from the nonlinear least-squares fit.

b Errors were calculated by standard error propagation. See SI Section 4 for details.

c Obtained from the two-set model.

d Obtained from the one-set model.

PEG adsorption may involve several factors that can give rise to cooperative effects, including chain connectivity, multiple contacts between the pore walls and the PEG chains, inter-PEG interactions, and intrapore diffusion. Although the one-set/two-set models provide a simple effective description of the adsorption events, they do not explicitly account for possible cooperative contributions. We therefore additionally examined the Fowler model^47^ as an exploratory model incorporating an occupancy-dependent interaction term to assess whether such cooperative effects could be resolved under different adsorption conditions (Table S4, see SI Section 4 for details). In this model, a term proportional to the fractional occupancy, *θ*, is added to the free energy, and the sign of its proportionality coefficient, *W*, is used to assess whether free-energy-based cooperativity is present, as Δ*G*(*θ*) = Δ*G*_0_ + 2*Wθ*, where Δ*G*_0_ is the standard free energy change for the one-set Langmuir model in the limit of low *θ*. Thus, negative *W* corresponds to favorable cooperative interactions.

For PEG adsorption in MeCN at 313 K, inclusion of the Fowler interaction term did not substantially alter the fitted adsorption parameters compared with the one-set model (Tables 1, 2 and S2–4). In addition, for the alcohol solvent systems, the Fowler model did not adequately reproduce the heat profiles. We therefore concluded that, as a first step, the simpler one-set/two-set models provide an appropriate basis for comparing the adsorption behavior among solvents. It should be noted that the successful application of the one-set/two-set models does not imply the existence of discrete binding site(s) in the present system. Rather, each set of apparent thermodynamic parameters can be regarded as an effective representation of an ensemble of potentially complex molecular processes underlying polymer adsorption.

From the ITC thermogram, important insight into host–guest stoichiometry can be obtained as the molar ratio between host and guest species by the fitting (Fig. 4).^28^ In this study, the host concentration, [MOF], was defined as the concentration of MOF unit cells in the measurement mixture, each unit cell being treated as an independent adsorption site. The guest concentration, [PEG], was defined as the concentration of PEG repeating units. Accordingly, the ratio [PEG]/[MOF] = *N* corresponds to the stoichiometric number of PEG repeating units accommodated per unit cell of 1. For 1, each unit cell accommodates approximately 3.1–4.6 PEG repeating units at full loading, as estimated from the reported maximum equilibrium adsorption amount (*q*_max_ = 0.2–0.3 g g^−1^), depending on the solvent used.^4^ In Fig. 4, the inflection point observed for PEG-H appeared around *N* = 1.92, 2.06, and 2.91, for PrOH, BuOH, and MeCN, respectively. These *N* values correspond to PEG adsorption amounts of *q* = 0.13, 0.13, and 0.19 g g^−1^, respectively (Table 1), which are in good agreement with the values calculated based on the reported equilibrium adsorption amounts (*q*_L_) in each solvent at the corresponding PEG concentrations of the adsorption endpoint (Table 1).^4^ However, the fitting for the EtOH data yielded an exceptionally high total stoichiometry of *N*_1_ + *N*_2_ = 6.37, exceeding the expected *q*_max_ of 1 (0.3 g g^−1^).^4^ This discrepancy indicates that the absolute stoichiometric parameters for EtOH are likely overestimated. Possible reasons include the relatively small magnitude of the adsorption heat, which can increase the correlation between *N* and Δ*H* in the two-set fitting, together with the relatively larger contribution from surface adsorption. In addition, the two-set model approximates the complex, adsorption-progress-dependent heat evolution in EtOH using two discrete components, which may further limit the quantitative accuracy of the fitted stoichiometries. Differences in adsorption conditions between the ITC and batch experiments may also contribute to the discrepancy.

### Solvent dependence of the thermodynamic parameters

The two-set model fitting for EtOH reveals distinct thermodynamic characteristics of the adsorption process. The fitting results showed two adsorption modes with distinct thermodynamic characteristics, denoted as Modes 1 and 2 (Table 1).^48^ Mode 1 adsorption was characterized by a small stoichiometry of *N*_1_ = 0.41, a large equilibrium constant of *K*_1_ = 2.66 × 10^3^ M^−1^, and an endothermic enthalpy change of Δ*H*_1_ = 4.79 kJ mol^−1^. In contrast, Mode 2 adsorption was characterized by a dominant capacity *N*_2_ = 5.96, a smaller equilibrium constant *K*_2_ = 0.35 × 10^3^ M^−1^, and a small exothermic enthalpy change of Δ*H*_2_ = −1.38 kJ mol^−1^ (Tables 1 and 2). The resulting parameters offer valuable insights into the unusual heat evolution observed in EtOH. In particular, the endothermic first component effectively captures the distinctive heat signal during the initial stage of adsorption, whereas the second component accounts for the subsequent exothermic adsorption process. One plausible interpretation is that the first component corresponds to PEG infiltration into initially PEG-free, EtOH-filled nanopores, while the second reflects adsorption into pores that have already been partially occupied by PEG. During the initial stage of adsorption, PEG must displace EtOH molecules from nanopores that are initially occupied exclusively by EtOH. Owing to the chemical similarity between EtOH and PEG repeating unit, this initial process is interpreted as being predominantly entropy-driven (described as Mode 1 adsorption in the two-set model). Once PEG has accumulated to some extent near the pore entrances, however, the adsorption becomes a more enthalpically favorable process because of possible inter-PEG interactions within the pores, which can be attributed to the second adsorption step (described as Mode 2 adsorption in the two-set model).

For the other solvents, on the other hand, the two-set model did not provide satisfactory fits. We attribute this to the absence of a large difference in the enthalpy changes between adsorption into PEG-free pores (Mode 1) and adsorption into pores already containing PEGs (Mode 2), making the two adsorption modes thermally indistinguishable. Based on the above interpretation, we used the parameters for Mode 2 adsorption in EtOH and those obtained from the one-set model fitting for the other solvents as the respective parameters for comparison across solvents (Fig. 5a).

**Fig. 5:**
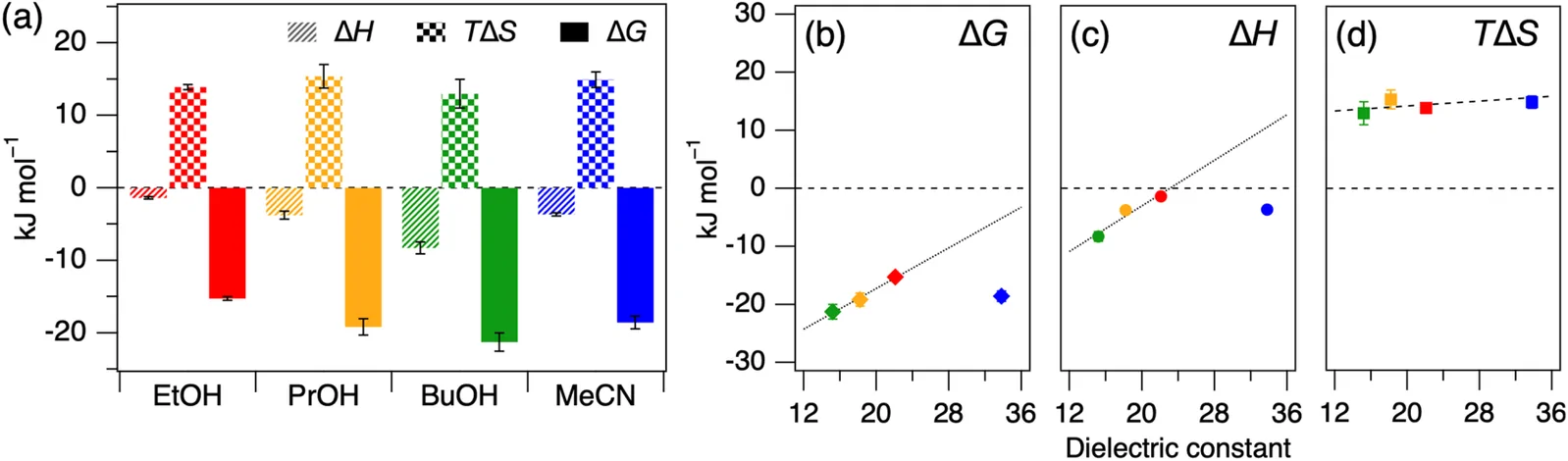
(a) Thermodynamic parameters, Δ*H*, *T*Δ*S*, and Δ*G*, obtained from the one-set/two-set model fitting for the given solvents. For EtOH, the parameters corresponding to Mode 2 adsorption, which exhibited the dominant adsorption capacity, are presented. (b–d) Thermodynamic parameters plotted as a function of solvent dielectric constant at 313 K for four solvents: EtOH (red), PrOH (orange), BuOH (green), and MeCN (blue). Dashed lines are a linear correlation among the alcohol solvents. Error bars represent standard uncertainties from fitting (Δ*H*) or error propagation (Δ*G* and *T*Δ*S*).

Based on the model fitting analysis, the association constants, *K*, were estimated at 313 K to be 0.35 × 10^3^ M^−1^ in EtOH, 1.58 × 10^3^ M^−1^ in PrOH, 3.55 × 10^3^ M^−1^ in BuOH, and 1.26 × 10^3^ M^−1^ in MeCN (Table 2). These *K* values are within the reasonable range of those reported for typical host–guest association and polymer adsorption onto solid surfaces in organic media.^31,49^

For the alcohol series, *K* increased with increasing alkyl chain length, corresponding to decreasing solvent polarity. A plot of Δ*G versus* the dielectric constant of the alcohols exhibited a linear correlation (Fig. 5b). This trend can be rationalized by considering the competitive nature of the adsorption process. PEG competes with solvent molecules for adsorption sites within the MOF pores. Given the relatively polar internal environment of 1, less polar solvents weaken MOF–solvent interactions and promote adsorption of the relatively polar PEG chains.^4^ In addition, solvent quality for PEG contributes to the observed affinity order. Poorer solvents favor PEG adsorption into the pores because confinement within the MOF channels provides a comparatively more stabilizing environment than the bulk solution.^4^ Similar correlations between solubility parameters and association constants have also been reported in other supramolecular host–guest systems.^31,32^

On the other hand, MeCN deviates from the dielectric trend observed for alcohols, despite exhibiting an intermediate *K* and Δ*G* value (Fig. 5b and Table 2). This discrepancy likely arises from additional factors beyond polarity, including differences in hydrogen-bonding ability, molecular size, and packing of solvent molecules within the confined pores. Because the channel of 1 is comparable in size to both PEG segments and solvent molecules, even subtle differences in their molecular structure can significantly influence adsorption thermodynamics.

The enthalpy changes also exhibited systematic solvent dependence within the alcohol series (Fig. 5a and Table 2). The magnitude of Δ*H* became increasingly negative with increasing alkyl chain length, namely Δ*H* = −1.38 kJ mol^−1^ for EtOH, −3.79 kJ mol^−1^ for PrOH, and −8.30 kJ mol^−1^ for BuOH. Thus, PEG infiltration from longer-chain/less polar alcohols is enthalpically more favorable. Interestingly, EtOH shows an exceptionally small enthalpic gain despite exhibiting pronounced PEG adsorption (Fig. 5a and Table 2). This observation is rationalized by recognizing that PEG infiltration necessarily involves counter-release of pre-adsorbed solvent molecules from the pores (Fig. 1). The experimentally observed Δ*H* therefore represents a net heat change composed of three contributions:Δ*H* = Δ*H*_MOF–PEG_ − Δ*H*_MOF–solvent_ − Δ*H*_PEG–solvent_

Because EtOH is structurally similar to the ethylene glycol repeating unit of PEG, the interactions of EtOH and PEG segments with the MOF interior are expected to be comparable. Consequently, Δ*H*_MOF–PEG_ and Δ*H*_MOF–solvent_ partially cancel under EtOH conditions, resulting in a small net enthalpy change (Δ*H* ∼ −Δ*H*_PEG–solvent_). In contrast, even small structural deviations in PrOH and BuOH alter this balance, likely producing significantly larger exothermic Δ*H* values. Variations in Δ*H*_PEG–solvent_ might also contribute to the overall solvent dependence. Using the thermodynamic relationship,Δ*G* = Δ*H* − *T*Δ*S* = −*RT* ln *K*

The entropy term *T*Δ*S* was also calculated for each solvent. Surprisingly, *T*Δ*S* was significantly positive in all adsorption-permitting solvents, namely 13.9 kJ mol^−1^ for EtOH, 15.4 kJ mol^−1^ for PrOH, 13.0 kJ mol^−1^ for BuOH, and 14.9 kJ mol^−1^ for MeCN at 313 K (Fig. 5a and Table 2). Moreover, the magnitude of *T*Δ*S* was nearly independent of solvent identity and larger than the enthalpic favorability |Δ*H*|. These results reveal a consistently favorable entropic contribution to PEG infiltration into 1. The large positive entropy contribution can be attributed to solvent release from the nanopores and PEG desolvation upon polymer insertion. In fact, batch adsorption experiments confirmed that the solvent molecules originally occupying the pores were displaced and replaced by PEG upon adsorption (Fig. S10 and SI Section 5 for the experimental details). Based on this result, the numbers of solvent molecules displaced per PEG repeating unit were estimated to be 1.03 ± 0.30, 0.52 ± 0.04, 0.34 ± 0.05, and 0.98 ± 0.02 for EtOH, PrOH, BuOH, and MeCN, respectively. These values are consistent with the expected values from the molecular structures of each solvent and the ethylene glycol repeating unit of PEG. Solvent molecules confined within the narrow pores experience restricted translational and rotational freedom;^50^ their release into bulk solution can yield an entropy gain that thermodynamically favors the adsorption process. A related contribution of solvent release and desolvation to favorable binding entropy has also been discussed for guest inclusion in a molecular cage in acetonitrile.^51^

The observed entropy change can be conceptually decomposed into contributions from solvation entropy (Δ*S*_solvation_) and configurational entropy (Δ*S*_config_).^31,44,52^ While polymer confinement incurs a negative Δ*S*_config_ due to conformational restriction, this penalty is outweighed by the large positive entropy change (namely, −Δ*S*_solvation_) associated with solvent displacement from MOF nanopores as well as PEG desolvation upon infiltration. Similar entropy-driven behavior has been reported in certain host–guest association^34,39,43^ and polymer adsorption systems,^44,49,53,54^ although it is less common in organic media.

Finally, to gain a more quantitative breakdown of the relative contributions of the thermodynamic parameters across solvents, Δ*H* and *T*Δ*S* were plotted against the dielectric constant as well (Fig. 5c and d). A plot of Δ*H* in the alcohol series also exhibited a linear correlation, with a significant deviation from the trend observed for MeCN (Fig. 5c), as seen in Δ*G* (Fig. 5b). Conversely, *T*Δ*S* did not show such a deviation for MeCN (Fig. 5d). These findings suggest that the solvent dependence of Δ*G* arises predominantly from differences in Δ*H* induced by the solvent properties. To explain the deviation in alcohols and MeCN quantitatively beyond solvent polarity alone, Kamlet–Taft analysis^55^ was employed (see SI Section 6). The results further suggested that, in addition to polarity, hydrogen-bonding ability makes a substantial contribution to the enthalpic favorability upon PEG infiltration (Table S5 and Fig. S11), which largely determines the magnitude of Δ*G* of the overall adsorption process.

### Balance between enthalpic and entropic contributions

To assess the enthalpy–entropy compensation (EEC) character of the present system, *T*Δ*S* was plotted against Δ*H* for the different solvent systems (Fig. S12). A linear correlation was observed with a slope of 0.20. A slope of unity corresponds to perfect compensation, where any changes in Δ*H* are fully offset by changes in Δ*S*, thus Δ*G* remains constant. The slope being much less than unity obtained here indicates weak compensation effects, suggesting that the solvent-dependent enthalpic changes are not largely balanced by the entropic effects. The positive intercept, *T*Δ*S*_0_ = 15.1 kJ mol^−1^ (Fig. S12) is comparable to reported values for inclusion complex formations (around 10–20 kJ mol^−1^).^31–33,56^ This positive intercept is consistent with the presence of a favorable entropic contribution that is relatively insensitive to solvent identity.^56^

In toluene, neither measurable heat transfer nor polymer adsorption was observed. The absence of adsorption may be attributed to strong interactions between toluene and MOF, potentially involving π–π interactions with the aromatic ndc ligands.^4^ This likely stabilizes toluene within the MOF pores in the bulk solution, thereby disfavoring the displacement of solvent molecules from the pores and from the polymer. As a result, the enthalpic stabilization associated with these interactions may outweigh the favorable MOF–polymer interactions and possible entropy gain, thereby preventing polymer infiltration.

These results demonstrate that solvent-dependent polymer adsorption in MOF nanopores is determined by a delicate balance among MOF–PEG, MOF–solvent, and PEG–solvent interactions. While enthalpy varies significantly with solvent identity, the large and nearly solvent-independent entropy gain promotes polymer infiltration thermodynamically.

### Molecular weight and temperature effects on the thermodynamic parameters

To gain deeper insight into the entropy-assisted adsorption process, the molecular weight and temperature dependences of the thermodynamic parameters were investigated using MeCN as the solvent. For the molecular weight dependence study, ITC measurements were performed for hydroxy-terminated PEGs with a molecular weight of 1 kg mol^−1^ (PEG1k) and 3 kg mol^−1^ (PEG3k) (Table S1) at 313 K. To examine the temperature effects, ITC measurements were performed for PEG-H at 308 K and 303 K. Adsorption thermograms for all samples were measured in the same manner (see SI Section 3), and the corresponding heat profiles were successfully fitted using the one-set model (Fig. S13a and b), enabling consistent extraction of thermodynamic parameters (Tables 2, S2 and S3).

The thermodynamic parameters for the PEGs with three different molecular weights (1, 2, and 3 kg mol^−1^) showed no significant difference within the studied MW region (Fig. S14). The observed molecular-weight independence of the enthalpy change is reasonable, as it indicates that the interaction mode of each PEG repeating unit remains essentially unchanged. For the entropy change, the solvation contribution per repeating unit, −Δ*S*_solvation_, is not expected to depend intrinsically on the overall chain length; therefore, the molecular-weight dependence should arise primarily from the configurational contribution, Δ*S*_config_. As an analogous conformational process, Δ*S*_config_ associated with PEG melting is known to exhibit little dependence on molecular weight when normalized per PEG repeating unit.^2,57^ It is therefore also reasonable that no clear molecular-weight dependence is observed in the net entropy change.

Accordingly, the free energy change per repeating unit, Δ*G*_RU_, may be regarded, to a first approximation, as independent of molecular weight. The total Δ*G* associated with adsorption of a polymer chain containing *n* repeating units can therefore be estimated as Δ*G*_total_ = *n* × Δ*G*_RU_. When Δ*G*_RU_ has a negative value, infiltration of the whole chain becomes thermodynamically more favorable as *n* increases.

Molecular weight dependence of thermodynamic parameters for PEG adsorption into 1 was also estimated in DMF using a liquid chromatography-based method.^4^ Although chromatographic retention itself does not directly reflect the fully equilibrated thermodynamics of the adsorption process, the reported trends nevertheless qualitatively provide valuable insights. Specifically, the apparent adsorption enthalpies (Δ*H*_app_) per PEG repeating unit were negative and almost constant across all examined molecular weights,^4^ consistent with the exothermic trends observed in the alcohol and MeCN systems in the present study. The magnitude of reported Δ*H*_app_ in DMF was relatively small (∼−1 kJ mol^−1^) compared with those observed in the current study, possibly due to stronger PEG–DMF and/or MOF–DMF interactions.

The temperature dependence of the thermodynamic parameters is summarized in Table 2 and Fig. 6. Upon decreasing the temperature, Δ*H* became more negative, indicating that adsorption becomes thermodynamically more favorable at lower temperatures. Although the change in Δ*H* was relatively modest, *T*Δ*S* exhibited a more pronounced temperature dependence, indicating that the entropy term (*T*Δ*S*) makes the larger contribution to the variation of the free energy change. Notably, *T*Δ*S* increased with decreasing temperature, leading to a larger positive *T*Δ*S* contribution and enhanced adsorption. This also means that Δ*S* itself increases as the temperature decreases. Thus, lowering the temperature promotes polymer adsorption both enthalpically and entropically. This behavior deviates from typical solution-phase reactions, where entropy contributions often increase with temperature.

**Fig. 6:**
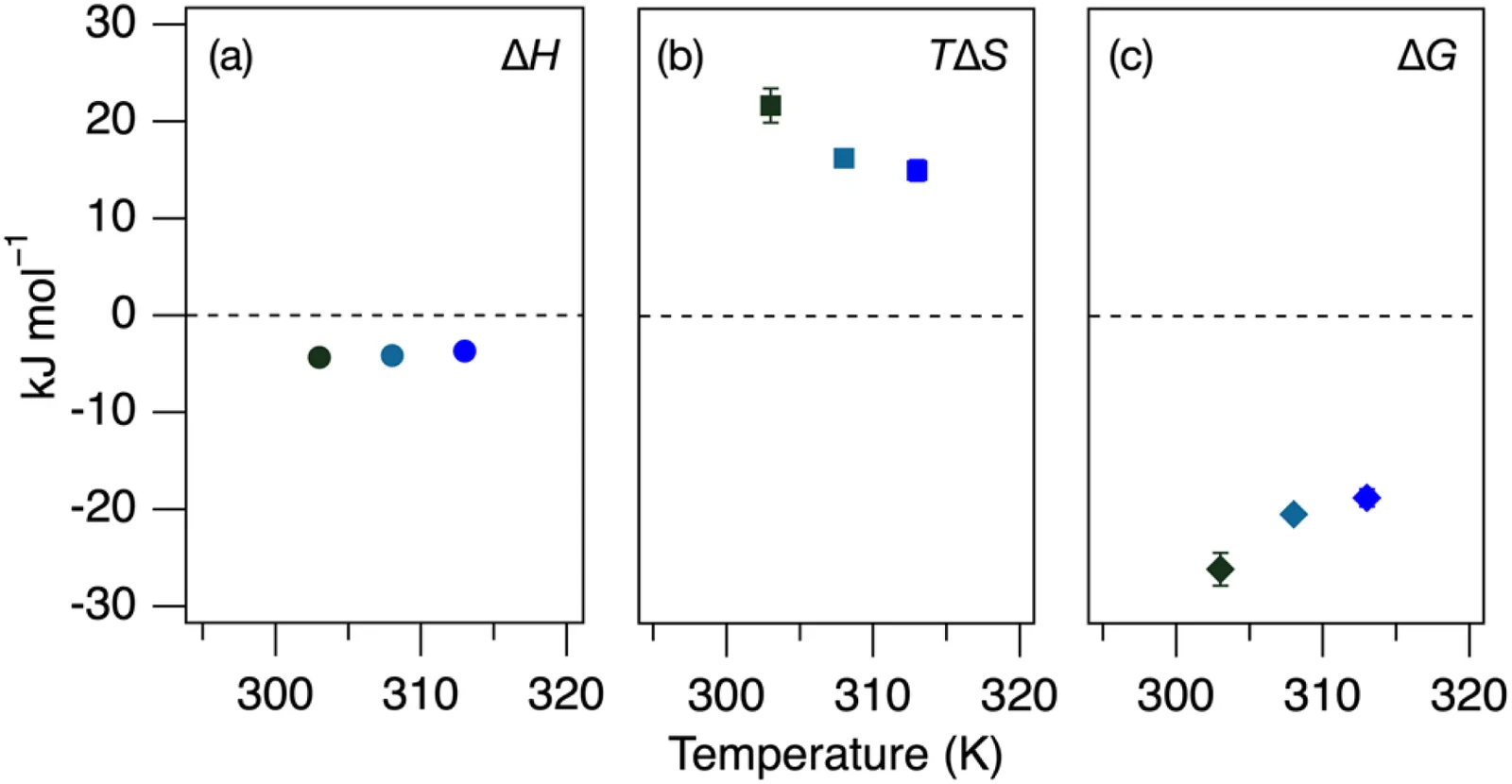
Temperature dependence of (a) Δ*H*, (b) *T*Δ*S*, and (c) Δ*G* for PEG-H adsorption into 1 in MeCN at 303, 308, and 313 K, obtained by the one-set model fitting. Error bars represent standard uncertainties from fitting (Δ*H*) or error propagation (Δ*G* and *T*Δ*S*).

One possible origin of this trend is a temperature-dependent change in the organization and release of solvent molecules confined within the pores and around PEG. At lower temperatures, solvent molecules confined within the MOF nanopores are expected to adopt more ordered and structured arrangements. Their release upon polymer infiltration, therefore, results in a larger entropy gain. In addition, desolvation of PEG chains in the bulk phase may contribute similarly, as solvent molecules surrounding the polymer become more structured at lower temperatures and yield greater entropy upon release during adsorption. These findings underscore the important role of solvents in modulating the thermodynamics of polymer adsorption into sub-nanometer pores under strong confinement. Further studies aimed at quantitatively elucidating the entropy contributions are currently underway.

Finally, we examined whether the cooperative contribution represented by the Fowler interaction parameter depends on PEG molecular weight and temperature (Fig. S13c, d and Table S4).^47^ At 313 K, the fitted *W* values remained close to zero across the PEG molecular weights examined, and no clear systematic molecular-weight dependence could be resolved. In contrast, for PEG-H, progressively more negative *W* values were obtained at lower temperatures (Table S4). This trend suggests that cooperative contributions to PEG adsorption may become more important at lower temperatures. Several effects may contribute to this behavior. For example, the slower adsorption at lower temperatures may provide greater opportunity for intra- and intermolecular interactions to develop. Alternatively, solvent molecules may adopt more highly structured arrangements within the pores at lower temperatures, and disruption and release of these structures upon replacement by PEG may become increasingly favorable as adsorption proceeds. The presence and extent of such cooperative effects, the conditions under which they emerge, and the corresponding molecular states within the pores will be investigated in greater detail and reported in future work.

### Kinetics analysis on ITC thermograms

Although ITC instruments are not generally designed for kinetic analysis, the heat-flow traces obtained during each titration inherently contain kinetic information associated with the adsorption process prior to reaching equilibrium.^58,59^ In this study, we sought to extract kinetic insight into PEG infiltration into MOF nanopores through quantitative analysis of the ITC thermograms.

In ITC measurements, the heat flow (d*Q*/d*t*) is recorded following each injection, and each peak typically exhibits a monotonic decay over time, reflecting the heat evolution associated with adsorption (Fig. 7a).^16,28,58^

**Fig. 7:**
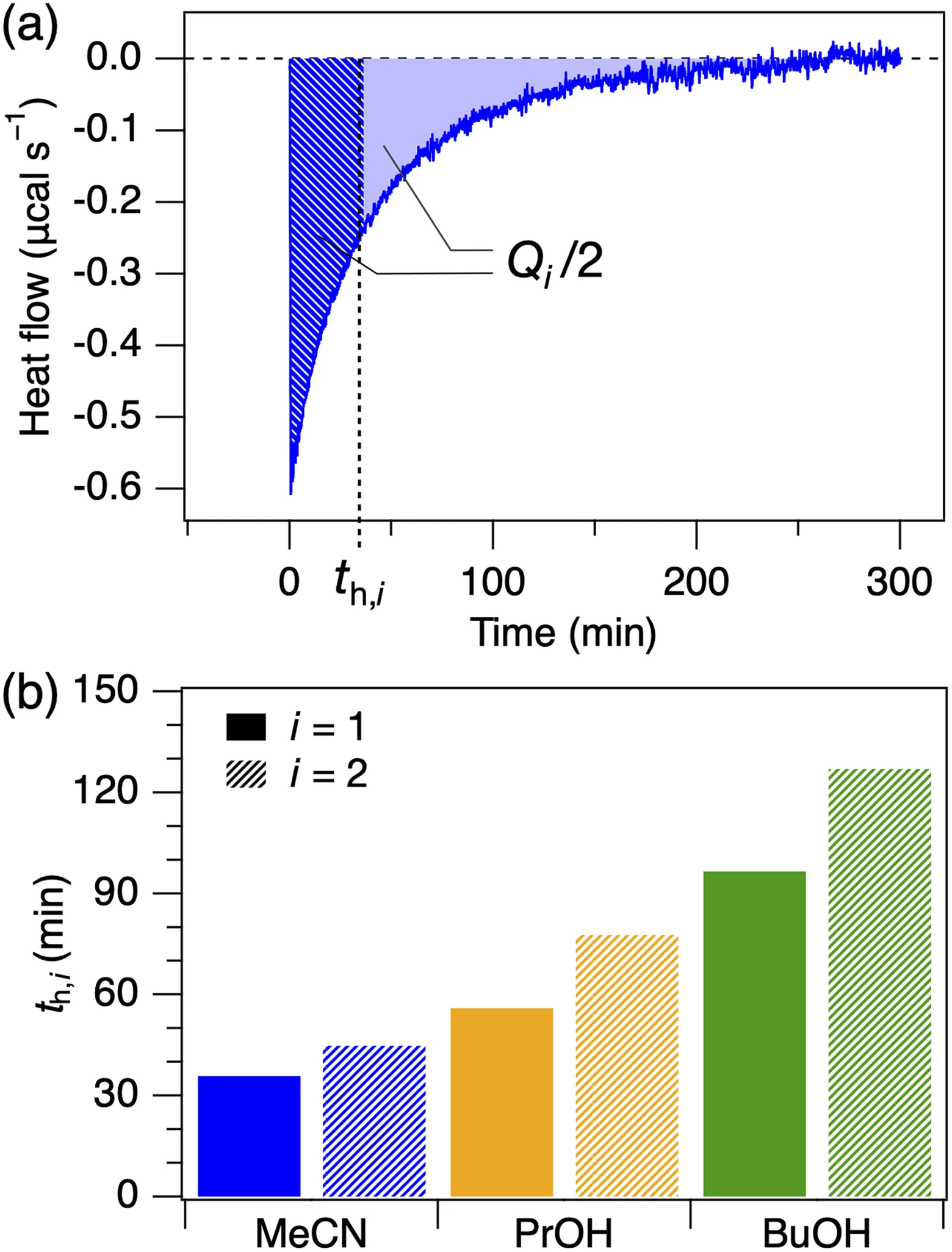
(a) Representative ITC thermogram for PEG adsorption into 1, measured in MeCN at 313 K. *t*_h,*i*_ is determined by the time required for the time-integrated heat to reach half of the total heat for *i*-th thermogram. (b) Solvent dependence of the time required for the time-integrated heat to reach half of the total heat for first and second thermograms, *t*_h,1_ (filled) and *t*_h,2_ (hatched), for PEG-H adsorption into 1 in MeCN (blue), PrOH (orange), and BuOH (green), respectively.

Assuming that the timescale of heat evolution reflects the progression of the adsorption-related process, we defined *t*_h,*i*_ as the time required for the cumulative heat of the *i*-th injection to reach half of the total integrated heat (*Q*_*i*_/2). It is important to note that the calorimetric response associated with PEG adsorption is sufficiently slow such that the instrumental response time in the order of seconds does not significantly affect the extracted characteristic timescales.^16^

Using this approach, the time required for the cumulative heat of the first and second injections to reach half of the total integrated heat, *t*_h,1_, at the initial stage and *t*_h,2_, at the early stage after partial pore occupation for the second injection were determined for the MeCN, PrOH, and BuOH systems to be *t*_h,1_/*t*_h,2_ = 35.8/44.8, 56.0/77.7, and 96.6/127 min, respectively, at 313 K, demonstrating a clear solvent dependence in the timescale of adsorption.

Interestingly, the heat-evolution timescale tended to increase with increasing solvent molecular size (Fig. 7b). This trend may indicate that diffusion and displacement of solvent molecules through the nanopores by the incoming PEG chains also become slower for larger solvent molecules, thereby affecting the overall adsorption kinetics. Because solvent molecules confined within the MOF pores must be displaced during polymer insertion, as mentioned earlier, their mobility directly influences the rate of infiltration. Larger size/higher viscosity solvents hinder this displacement process, thereby slowing adsorption.

Additionally, it is noteworthy that *t*_h,2_ showed a larger value than *t*_h,1_ for all cases, indicating that adsorption became slower as PEG loading progressed (Fig. 7b). This trend can be attributed to the inherently slow diffusion of macromolecules under nanoscale confinement.^46^ Pre-adsorbed PEG chains hinder subsequent infiltration of incoming chains due to restricted mobility and single-file-like diffusion within the pseudo-one-dimensional channels.

The temperature- and molecular-weight-dependent measurements also showed that adsorption proceeded more rapidly at higher temperatures and for lower-molecular-weight PEGs (Fig. S15). These trends suggest that the mobility and diffusion of solvent molecules and polymer chains within and around the nanopores are reflected in the rate of adsorption progression, consistent with the above interpretation.

These findings provide insight into the dynamics associated with polymer infiltration and suggest that both displacement/release of solvent molecules from the nanopores and polymer diffusion within the nanopores contribute to the adsorption kinetics. Together with the thermodynamic analysis, these results highlight the dual role of solvent molecules in controlling both the energetics and dynamics of polymer adsorption in MOF nanopores.

## Conclusions

We elucidated the thermodynamic and kinetic characteristics of polymer adsorption into sub-nanometer MOF nanopores from solution. Isothermal titration calorimetry across multiple organic solvents and the Langmuir-model-derived thermodynamic parameters revealed a consistently favorable entropic contribution to polymer infiltration, despite the expected conformational entropy loss under confinement. The entropy gain was large and nearly independent of solvent identity, whereas the enthalpy varied significantly, indicating that solvent expulsion from the nanopores and PEG desolvation are plausible contributors to the adsorption driving force. Thermodynamic analysis further highlighted the importance of the interplay among MOF–polymer, MOF–solvent, and polymer–solvent interactions. Solvent-dependent enthalpic contributions, including the solvent affinity for the MOF pores and the polymer solubility to the solvent, primarily determine whether adsorption is thermodynamically favorable or not.

Further analysis of the heat profiles using the Fowler model suggested that cooperative adsorption effects may emerge under certain conditions, depending on factors such as the solvent and temperature. While the thermodynamic parameters obtained here are inherently model-dependent, the simplified models employed provide a useful framework for understanding polymer adsorption into MOF nanopores. Developing new models that explicitly account for the molecular complexity of the nanopore-threading adsorption systems remains an important challenge for future studies.

Thermogram-based kinetic analysis revealed that the adsorption rate is likely influenced by solvent molecular size and decreases as PEG adsorption proceeds. These findings suggest that solvent mobility and nanoscale confinement effect influence the dynamics of polymer infiltration.

It should be noted that the pore size and even the pore dimensionality of MOFs can be decisive factors governing both the thermodynamics and kinetics of polymer adsorption. Future studies will incorporate these structural factors to establish a more comprehensive understanding of the energy landscape governing polymer adsorption in MOFs.

This work provides guiding principles for predicting and controlling polymer infiltration in confined spaces. These insights will advance the rational design of MOF-based systems for polymer separation, recognition, and nanocomposite fabrication, and are expected to be broadly applicable to adsorption processes involving macromolecules in highly confined environments.

## Author contributions

Conceptualization: N. H., T. U., methodology: Y. K., N. H., investigation: Y. K., N. H., data acquisition: Y. K., data curation: Y. K., N. H., supervision: N. H., T. U., writing – original draft: Y. K., N. H., writing – review & editing: N. H., T. U.

## Conflicts of interest

There are no conflicts to declare.

## Supplementary Material

SC-OLF-D6SC03917K-s001

## Data Availability

All the data supporting the findings of this study are available in the article and the supplementary information (SI). Supplementary information is available. See DOI: https://doi.org/10.1039/d6sc03917k.
