# Supplementary material for: What drives polymer adsorption into MOF nanopores? Insights into the role of solvents from titration calorimetry
Source: Chem Sci. 2026 Oct 19. Online ahead of print. doi: 10.1039/d6sc03917k (PMC13752725; doi:10.1039/d6sc03917k)
Supplement: Supplementary file 1 [file SC-OLF-D6SC03917K-s001.pdf]

Supplementary Information (SI) for Chemical Science.
This journal is © The Royal Society of Chemistry

Electronic Supplementary Information

What drives polymer adsorption into MOF nanopores? Insights into the role of solvents from titration calorimetry

Yu Kono, Takashi Uemura* and Nobuhiko Hosono*

1. General Instruments

Powder X-ray diffraction (PXRD) data were recorded on a Rigaku model SmartLab X-ray diffractometer using Cu Kα radiation.

Particle size distribution analysis was performed using a HORIBA LA-950 laser scattering particle size distribution analyzer, with the samples being dispersed in acetone.

Scanning electron microscopy (SEM) measurements were performed using a Hitachi model SU-5000 at an accelerating voltage of 15 kV. Samples were deposited on a conducting carbon tape attached on a SEM sample holder, then coated with osmium.

N_2 sorption isotherm was measured using MicrotracBEL BELSORP-mini. The sample was activated at 120°C under vacuum for 6 h prior to measurement.

Analytical size-exclusion chromatography (SEC) measurements were performed using two polystyrene gel columns in series (Shodex KF-806M) at 40 °C on a SHIMADZU model LC-2050 system equipped with a refractive index (RI) detector. The mobile phase was tetrahydrofuran (THF) at a flow rate of 1.0 mL min^{-1}.

^{1}H nuclear magnetic resonance (NMR) spectra in solution were recorded using a JEOL JNM-ECZL500R 500 MHz spectrometer, and the data were analyzed using Bruker Topspin 4.5.0.

Isothermal Titration Calorimetry (ITC) measurements were conducted using a VP-ITC titration microcalorimeter (Malvern, MicroCal LLC.). The sample cell, made of Hastelloy C-276, had an effective cell volume of V_0 = 1.458 mL. The data analyses, including baseline correction, integration, and fitting to the conventional Langmuir-type models, were performed on the dedicated application from MicroCal, based on Origin 7.0. Further analyses using additional fitting models were implemented and performed in Python 3.11.

2. Materials

All reagents and chemicals used in this study were obtained from FUJIFILM Wako Pure Chemicals, Tokyo Chemical Industry (TCI), or Sigma-Aldrich. Trityl-terminated polyethylene glycol (**PEG-Tr**) was synthesized from hydroxy-terminated PEG with a molecular weight of 2 kg mol^{-1} (**PEG-H**) and trityl chloride by following a procedure described in the literature.[S1]

Synthesis of 1.

Powdery $[Zn_2(ndc)_2(ted)]_n$ (**1**) was synthesized according to the previously reported procedure with a slight modification.[S2] *N*,*N*-dimethylformamide (DMF, 80 mL) solution of $Zn(NO_3)_2 \cdot 6H_2O$ (2.28 g, 7.65 mmol), 1,4-naphthalenedicarboxylic acid (ndc, 1.65 g, 7.65 mmol) and triethylenediamine (ted, 0.43 g, 3.83 mmol) was stirred vigorously at 120 °C for 48 h. The reaction mixture was then cooled to room temperature, and the product was collected by suction filtration. The white powder thus obtained was washed with DMF (ca. 50 mL × 3), affording powdery **1** with DMF as the guest solvent (ca. 5 g). **1** was evacuated under vacuum at 120°C for 6 h prior to use.

Synthesis of single crystals of 1.

Single crystals of **1** were synthesized according to the procedure described above with a slight modification.[S2] DMF (80 mL) solution of $Zn(NO_3)_2 \cdot 6H_2O$ (2.28 g, 7.65 mmol), ndc (1.65 g, 7.65 mmol) and ted (0.43 g, 3.83 mmol) was stirred vigorously at room temperature for 1 h. The reaction mixture was then centrifuged to remove the kinetically produced powder, and the supernatant was further filtered out using 0.45 μm PTFE membrane filter to collect a clear solution mixture. The resulting solution was divided into vials and then heated at 100 °C for 2 days in an oven under static conditions. The millimeter-sized brown cube thus obtained was washed with DMF (ca. 50 mL × 3) and stored under pure DMF.

3. Isothermal Titration Calorimetry

Alcohols and acetonitrile were degassed for 10 min using a ThermoVac system (Malvern, MicroCal LLC.) prior to use. **1** was activated at 120°C under vacuum to remove DMF from the pores. After activation, the desired volume of solvent was carefully added, and the suspension was sonicated for 5–10 min to obtain a homogeneous dispersion. PEG solutions were sonicated at room temperature to ensure complete dissolution. The concentrations and injection volumes were optimized for each solvent according to the solubility of PEG, as summarized in Table S6. The reference power was set to 3, 5 or 10 μcal s^{-1}.

Prior to measurement, both the sample cell and reference cell were filled with the corresponding solvent and equilibrated at (measurement temperature − 2) K for sufficient time to stabilize the baseline. The solvent in the sample cell was then replaced with the metal–organic framework (MOF) suspension, whereas the solvent alone was used for blank experiments. The temperature was subsequently increased to the measurement temperature, followed by a pre-stirring equilibration step.

The injection syringe was thoroughly rinsed and finally washed with the measurement solvent before being filled with the PEG solution. The syringe was then inserted into the sample cell, and stirring was initiated at a constant rate of 307 rpm. After a final baseline equilibration period, a preliminary injection (~20% of the standard titration volume) was performed as a dummy injection to reduce artifacts commonly associated with the initial injection. The heat from this preliminary injection was therefore excluded from subsequent data analysis. Injection intervals were adjusted for each solvent to ensure that equilibrium was reached after each injection. The maximum interval periods are summarized in Table S6. Heat flow was recorded as a function of time throughout the experiment.

All comparative measurements for the five solvents, ethanol (EtOH), 1-propanol (PrOH), 1-butanol (BuOH), acetonitrile (MeCN), and toluene, were conducted at 313 K. For molecular weight- or temperature-dependent studies, MeCN was selected as the representative solvent. Additional measurements were performed using PEGs with a molecular weight of 1 kg mol^{-1} (PEG1k) and 3 kg mol^{-1} (PEG3k) at 313 K for the former, and **PEG-H** at 303 K and 308 K for the latter, following the same procedure.

4. Fitting Analysis

General procedure.

The raw heat-flow curves were integrated over time to obtain the normalized heat per injected amount of PEG. The resulting heat profiles were plotted as a function of the molar ratio in the sample cell, [PEG]/[MOF], where [PEG] represents the molar concentration of PEG repeating units and [MOF] represents the molar concentration of MOF unit cells.

The resulting heat profiles were analyzed using several binding models: the one-set, two-set, and Fowler cooperative adsorption model,[S3] to find the model that describes the experimental results well. Data points that exhibited significant baseline drift or noise, likely arising from instrumental disturbances such as vibration or air bubbles, were excluded from the fitting analysis. The heat of dilution of PEG, obtained from blank measurements, was subtracted from the corresponding heat profiles.

The association constant (K), binding stoichiometry (N), and enthalpy change (ΔH) were obtained as model-dependent fitting parameters by nonlinear least-squares fitting of each ITC dataset to the respective adsorption model. The Gibbs free energy change (ΔG) and entropy contribution ($T\Delta S$) were thus calculated from the fitted K and ΔH values according to

$$\Delta G = -RT\ln K$$

and

$$T\Delta S = \Delta H - \Delta G = \Delta H + RT\ln K$$

where R is the gas constant, and T is the absolute measurement temperature.

The reported uncertainties of the directly fitted parameters represent standard errors estimated from the covariance matrix of the nonlinear least-squares fit. For the derived thermodynamic quantities, standard errors were calculated by first-order error propagation using the covariance matrix of the fitted parameters. Accordingly, neglecting the uncertainty in temperature,

$$\sigma_{\Delta G} = \frac{RT}{K}\sigma_K$$

and

$${\sigma_{T\Delta S}}^2 = {\sigma_{\Delta H}}^2 + {\sigma_{\Delta G}}^2 + 2\frac{RT}{K}\mathrm{Cov}(K, \Delta H)$$

Here, $\sigma_K, \sigma_{\Delta H}, \sigma_{\Delta G}$, and $\sigma_{T\Delta S}$, denote the standard errors of K, ΔH, ΔG, and $T\Delta S$, respectively, and Cov(K, ΔH) denotes the covariance between the fitted K and ΔH parameters.

Fitting analysis using the Fowler model.

Here, we define the variables and parameters as follows:

K_F = low-coverage association constant;

W = cooperative interaction parameter;

θ_i = fractional occupancy of the adsorption capacity after i-th injection;

N = stoichiometry;

V_0 = the active cell volume;

v = injection volume;

ΔH = adsorption enthalpy change per mole of adsorbed PEG repeating unit;

C_0 = PEG repeating unit concentration initially filled into the injection syringe;

X_i = total PEG repeating unit concentration after i-th injection;

M_i = total MOF unit cell concentration after i-th injection.

The ITC data were analyzed using the Fowler adsorption model[S3] as an extension of the one-set Langmuir type model, implemented in Python as described below. The procedure followed the method used in the MicroCal Origin software for the one-set/two-set model fitting. The concentration lists of M_i and X_i were calculated by the software and exported for subsequent analysis.

The free PEG repeating unit concentration at equilibrium after i-th injection was calculated from the mass balance as

$$[\mathrm{PEG}]_{\mathrm{free},i} = X_i - NM_i\theta_i$$

For each injection state, θ_i was obtained by numerically solving

$$K_F = \frac{\theta_i}{(1-\theta_i)[\mathrm{PEG}]_{\mathrm{free},i}} \exp\left(\frac{2W\theta_i}{RT}\right)$$

The solution was restricted to the physically allowed range,

$$0 < \theta_i < \min\left(1, \frac{X_i}{NM_i}\right)$$

The cumulative adsorption heat in state i was then calculated as

$$Q_i = NM_i\theta_i V_0 \Delta H$$

The heat generated by the i-th injection, corresponding to the transition from state $i-1$ to state i, was calculated using the volume-displacement correction:

$$\Delta Q_i^{\mathrm{pred}} = Q_i - Q_{i-1} + \frac{v}{V_0}\frac{Q_i + Q_{i-1}}{2}$$

The predicted normalized heat for the i-th injection, NDH_i^{pred}, was then obtained as

$$NDH_i^{\mathrm{pred}} = \frac{\Delta Q_i^{\mathrm{pred}}}{C_0 v} = \frac{\Delta Q_i^{\mathrm{pred}}}{n_{\mathrm{inject}}}$$

where n_{inject} is the molar amount of PEG repeating units introduced in the i-th injection.

The parameters K_F, N, ΔH, and W were determined by nonlinear least-squares minimization of the residual sum of squares between the predicted, NDH_i^{pred}, and experimentally observed

normalized heats, NDH_i^{obs}. To improve numerical scaling during optimization, K_F was reparameterized as $\log_{10}(K_F/\text{M}^{-1})$.

The fitting procedure then involved: (1) manual specification of initial estimates for K_F, N, ΔH, and W (which are based on the fitted parameters by the one-set model); (2) calculation of NDH_i^{pred} for each injection and comparison of these values with the experimentally observed normalized heat, NDH_i^{obs}; (3) refinement of $\log_{10}(K_F/\text{M}^{-1})$, N, ΔH, and W using the MIGRAD algorithm implemented in the iminuit package for Python; and (4) iteration of the above procedure until no further significant improvement in the fit was obtained. Standard uncertainties of the fitted parameters were obtained from the covariance matrix evaluated using HESSE algorithm and scaled by the residual variance.

5. Verification of the solvent release upon PEG adsorption

The ITC results suggest that the large positive entropy change predominantly contributes to the PEG infiltration into the MOF nanopores, which can be attributed primarily to solvent release from the nanopores. To verify that the pore-confined solvent was displaced by PEG upon infiltration event, we performed the quantitative analysis by ^{1}H solution NMR using both the MOF filled only with each solvent (EtOH, PrOH, BuOH, and MeCN) and the corresponding MOF–PEG composites prepared by adsorbing PEG from those solvents. The amounts of retained solvent and adsorbed PEG were then estimated from the NMR data.

Single crystals of **1** had been solvent-exchanged from DMF to BuOH and stored in BuOH, which were activated under vacuum at 120 °C for 6 h prior to use. Several activated crystals were immersed separately in EtOH, PrOH, or MeCN and left for 16 h at 25 °C. Two crystals filled exclusively with each solvent were then taken, placed on filter paper, and gently blotted to remove residual solvent from the external surface before NMR analysis.

PEG adsorption experiments were performed as follows. Two activated crystals of **1** (ca. 20 mg) were placed in a vial, and 4 mL of a **PEG-H** solution (2.5 mg mL^{-1}) was added. Five sets of the vials were prepared for each solvent. Adsorption was then allowed to proceed at 40 °C. After predetermined periods of 24, 48, 72, 120, or 168 h, the crystals were removed from the solution, placed on filter paper, and gently blotted to remove residual solution from the external surface before NMR analysis.

For NMR measurements, the crystals were dispersed in approximately 0.8 mL of a 9:1 (v/v) mixture of dimethyl sulfoxide-d_6 and a deuterated acid. As an acid to decompose the MOF framework, either trifluoroacetic acid-*d* or deuterium chloride was used. In all cases, the crystals were completely dissolved by sonication. The amounts of adsorbed PEG, $q(t)$, and retained solvent, $r(t)$, per gram of MOF at time t were calculated from the integral ratios of the PEG and solvent resonances, respectively, to those of the ndc ligand (Fig. S10). Specifically, as the initial solvent-filled state, $r(0)$ was defined as the mean of three independent measurements.

The number of solvent molecules displaced per PEG repeating unit at time t, $s(t)$, was then calculated as

$$s(t) = \frac{\{r(0) - r(t)\}/M_{\mathrm{S}}}{q(t)/M_{\mathrm{P}}}$$

where M_{S} and M_{P} denote the molar mass of each solvent and PEG repeating unit (–CH_2CH_2O–), respectively.

6. Fitting the thermodynamic parameters by Kamlet-Taft parameters

Plots of the fitted ΔG and ΔH values against the solvent dielectric constant showed linear relationships among the alcohols, whereas MeCN deviated substantially from these trends (Fig. 5b and c). In contrast, $T\Delta S$ values were nearly identical within the four solvents (Fig. 5d). To investigate the origin of the deviation of ΔG and ΔH more quantitatively, we employed the Kamlet–Taft solvent parameters, which describe solvent properties beyond polarity alone.[S4]

The thermodynamic parameters ($Y = \Delta G$, ΔH, or $T\Delta S$) were separately fitted using the linear model,

$$Y = Y_0 + s\pi^* + a\alpha$$

using Kamlet-Taft parameters, π^*, an empirical index of solvent dipolarity/polarizability, and α, a scale of hydrogen-bond donor abilities. Reported values of π^*/α are as follows: 0.54/0.83 for EtOH, 0.52/0.78 for PrOH, 0.47/0.79 for BuOH, and 0.75/0.19 for MeCN.[S4] Y_0 is the intercept, while s and a are the coefficients for π^* and α, respectively. Linear regression provided the fitting values, Y_0, s and a (Table S5). The resulting models provided a descriptive representation of the four-solvent dataset (Fig. S11). The fitted coefficients (s and a) for ΔG and ΔH were approximately comparable in magnitude, whereas those for $T\Delta S$ were much smaller and closer to zero (Table S5). This result suggests that the solvent dependence captured by π^* and α is expressed predominantly in ΔH and is consequently reflected in ΔG, whereas no clear dependence of $T\Delta S$ on these solvent parameters was observed. This interpretation is also consistent with the solvent independence of the entropy term discussed in the main text.

7. Supporting Tables

Table S1. SEC data of the hydroxy-terminated PEGs used in this study. Standard PEGs were used for molecular weight calibration.

Code	M_n (g mol^{-1})	M_W (g mol^{-1})	*Đ*
PEG1k	960	1000	1.05
**PEG-H** (PEG2k)	1940	2010	1.04
PEG3k	2740	2840	1.03

Table S2. Stoichiometric values *N* obtained from the one-set model fitting and equilibrium adsorption amounts of PEGs into **1** in MeCN with different molecular weights and different temperatures.

Code	Temperature (K)	N^a ([PEG]/[MOF])	q^b (g g^{-1})
**PEG-H**	313	2.91 ± 0.14	0.19
**PEG-H**	308	2.85 ± 0.05	0.19
**PEG-H**	303	2.94 ± 0.03	0.19
PEG1k	313	2.53 ± 0.18	0.17
PEG3k	313	3.01 ± 0.06	0.20

[a] Determined through ITC measurements and fitting to the one-set model. Errors represent the fitting uncertainties obtained from the nonlinear least-squares fit.
[b] PEG adsorption amount corresponding to *N*.

Table S3. Molecular weight dependence on *K* and thermodynamic parameters obtained from fitting to the one-set model. All measurements were conducted at 313 K in MeCN.

Code	K^a (10^3 M^{-1})	ΔG^b (kJ mol^{-1})	ΔH^a (kJ mol^{-1})	$T\Delta S^b$ (kJ mol^{-1})
PEG1k	1.14 ± 0.56	−18.3 ± 1.3	−3.53 ± 0.34	14.8 ± 1.5
PEG3k	0.84 ± 0.10	−17.5 ± 0.3	−3.65 ± 0.09	13.9 ± 0.4

[a] Errors represent the fitting uncertainties obtained from the nonlinear least-squares fit.
[b] Errors were calculated by standard error propagation.

Table S4. Fitting parameters obtained from the Fowler adsorption model fitted to the heat profiles measured in MeCN. K_F: low-coverage association constant, W: cooperative interaction parameter. Negative W indicates cooperative interactions.

Injected Sample	Temp. (K)	N^a ([PEG]/[MOF])	$\log_{10}(K_F/\mathrm{M}^{-1})^a$	ΔH^a (kJ mol^{-1})	W^a (kJ mol^{-1})
**PEG-H**	313	2.90 ± 0.28	3.07 ± 0.52	−3.69 ± 0.41	−0.08 ± 1.50
**PEG-H**	308	2.66 ± 0.18	2.83 ± 0.38	−4.43 ± 0.34	−1.64 ± 0.90
**PEG-H**	303	2.87 ± 0.08	2.60 ± 0.25	−4.50 ± 0.25	−4.77 ± 1.30
PEG1k	313	2.51 ± 0.60	3.02 ± 1.12	−3.55 ± 0.88	−0.10 ± 3.29
PEG3k	313	3.03 ± 0.30	2.96 ± 0.45	−3.62 ± 0.36	0.11 ± 1.42

[a] Errors represent standard uncertainties estimated from the HESSE covariance matrix and scaled by the residual variance.

Table S5. Results of Kamlet-Taft analysis. Kamlet-Taft parameters: π^*, an index of solvent dipolarity/polarizability; α, a scale of hydrogen-bond donor abilities. Y_0 is the intercept, s and a are the coefficients for π^* and α, respectively, of the equation $Y = Y_0 + s\pi^* + a\alpha$.

Dependent variable Y	$Y_0{}^a$	s^a	a^a
ΔG	−75.8 ± 12.5	69.3 ± 15.1	27.3 ± 6.1
ΔH	−70.8 ± 8.39	81.7 ± 10.2	30.8 ± 4.11
$T\Delta S$	5.0 ± 21	12.4 ± 25.4	3.4 ± 10.3

[a] Errors represent fitting uncertainties.

Table S6. Parameters for the titration experiments.

Solvent	Injected Sample	Temp. (K)	M_0[a] (mM)	C_0[b] (mM)	Injected amount v (μL)	Maximum injection interval (h)
EtOH	**PEG-H**	313	4.3	269	10	2
EtOH	**PEG-Tr**	313	4.3	221	15	0.5
PrOH	**PEG-H**	313	1.7	91	10	8
BuOH	**PEG-H**	313	1.7	68	12	8
MeCN	**PEG-H**	313	4.1	182	10	6
MeCN	**PEG-H**	308	4.1	182	10	7
MeCN	**PEG-H**	303	4.1	182	10	10
MeCN	PEG1k	313	4.1	182	10	4
MeCN	PEG3k	313	4.1	181	10	9
Toluene	**PEG-H**	313	4.1	227	10	2

[a] Concentration of the MOF unit cell initially filled into the sample cell, calculated based on the molar mass of [$Zn_2(ndc)_2(ted)$] = 671.3.

[b] Concentration of the PEG repeating unit initially filled into the injection syringe, calculated based on the molar mass of (–CH_2CH_2O–) = 44.05.

8. Supporting Figures

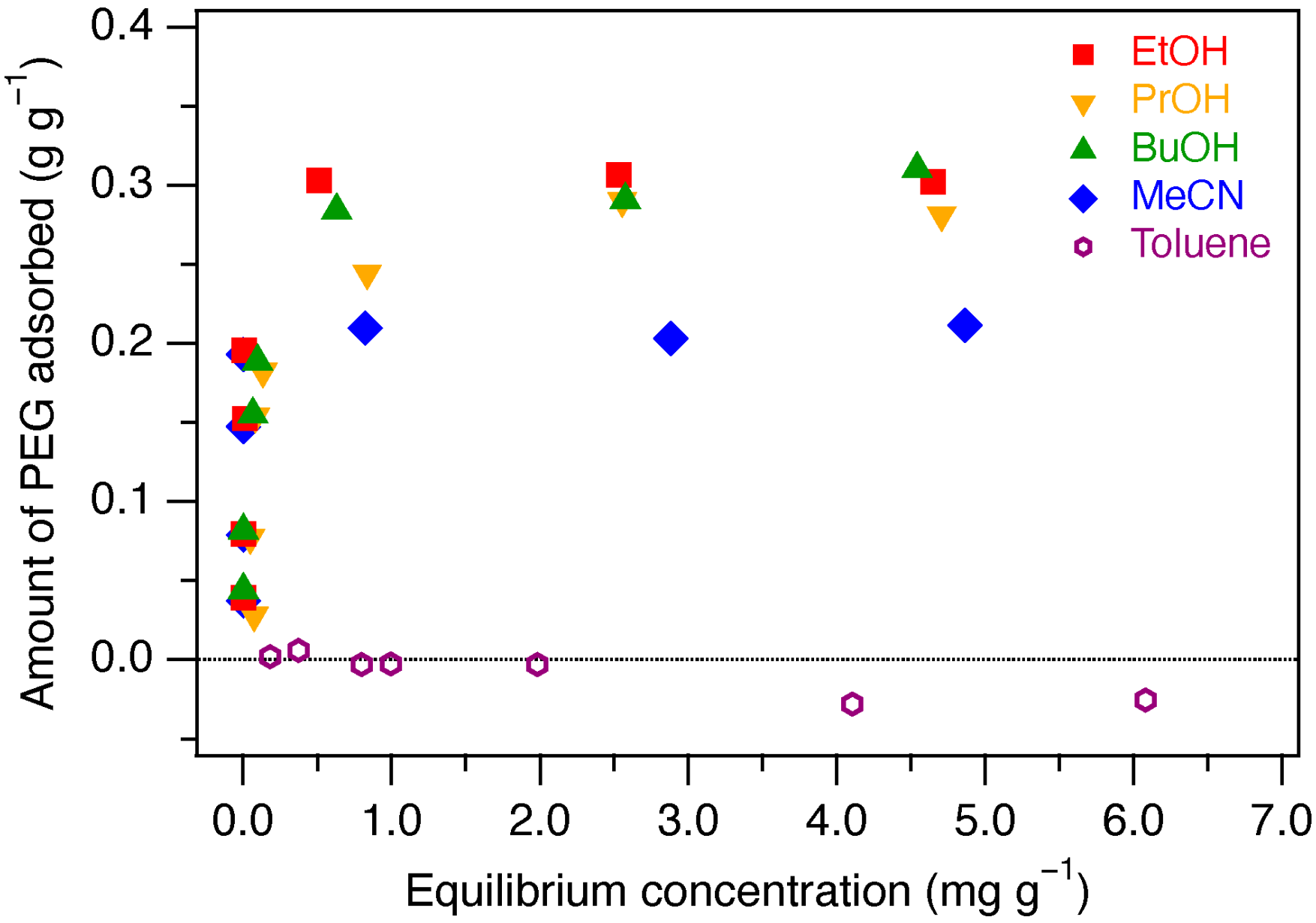

Fig. S1. Adsorption isotherms of **PEG-H** into **1** in the given solvents at 313 K, showing pronounced solvent-dependent adsorption behavior. Reproduced from the previous report.[S5]

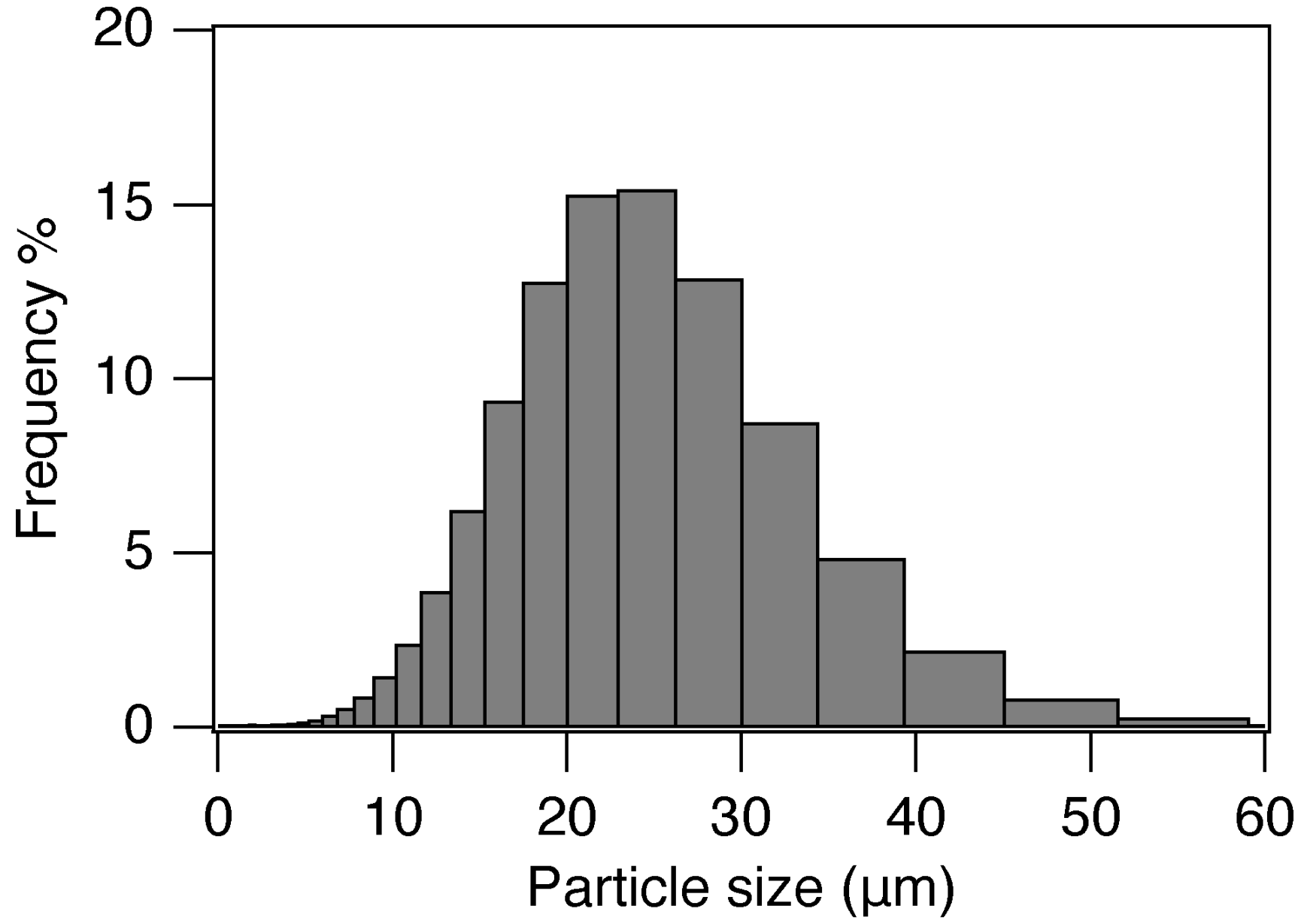

Fig. S2. Particle size distribution of **1** used for the ITC measurements. The sample was dispersed in acetone and measured at 25 °C. The average particle size was 19.7 ± 7.2 µm.

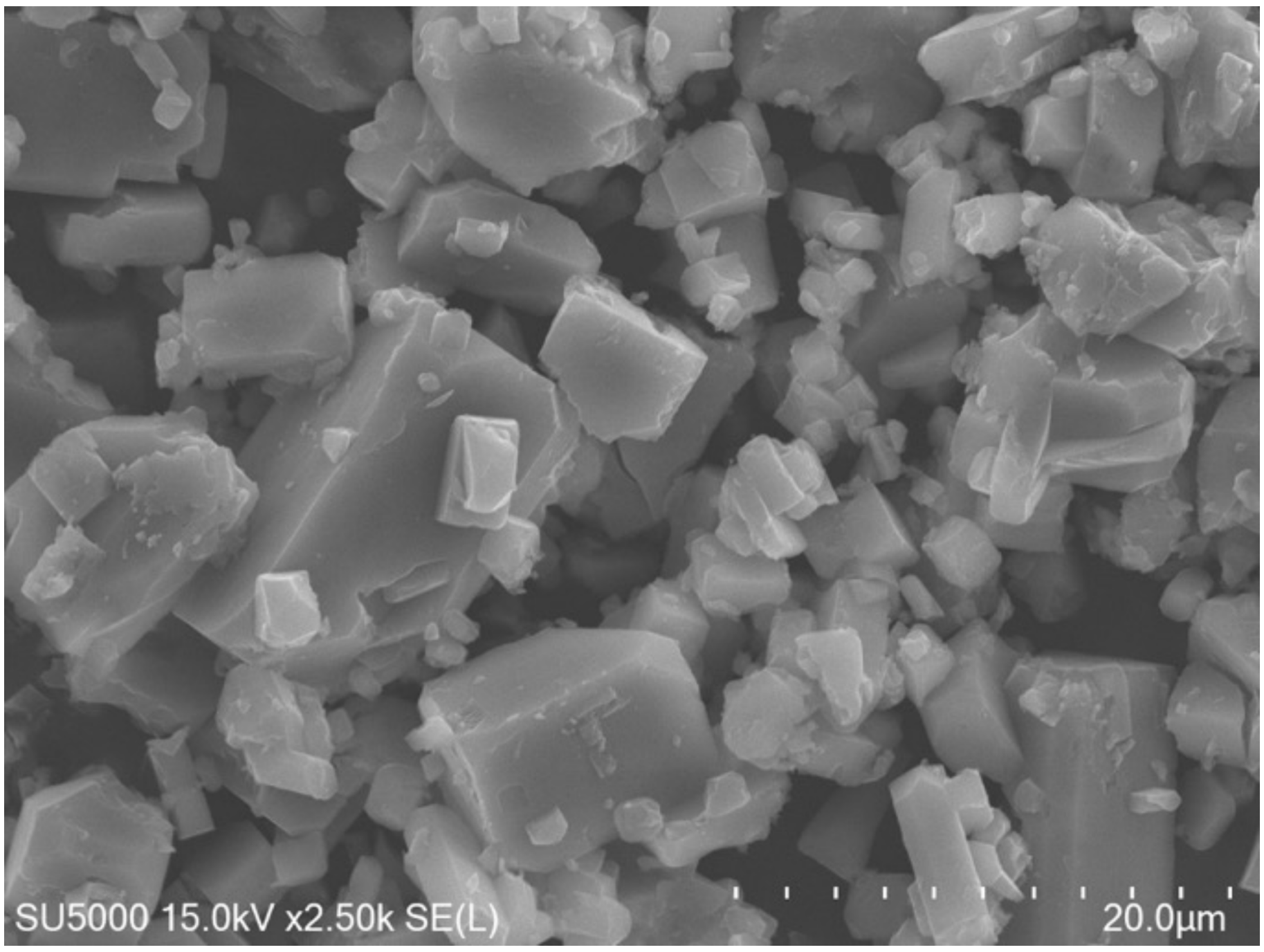

Fig. S3. A SEM image of the particles of **1** used for the ITC measurements, showing their cuboid morphology.

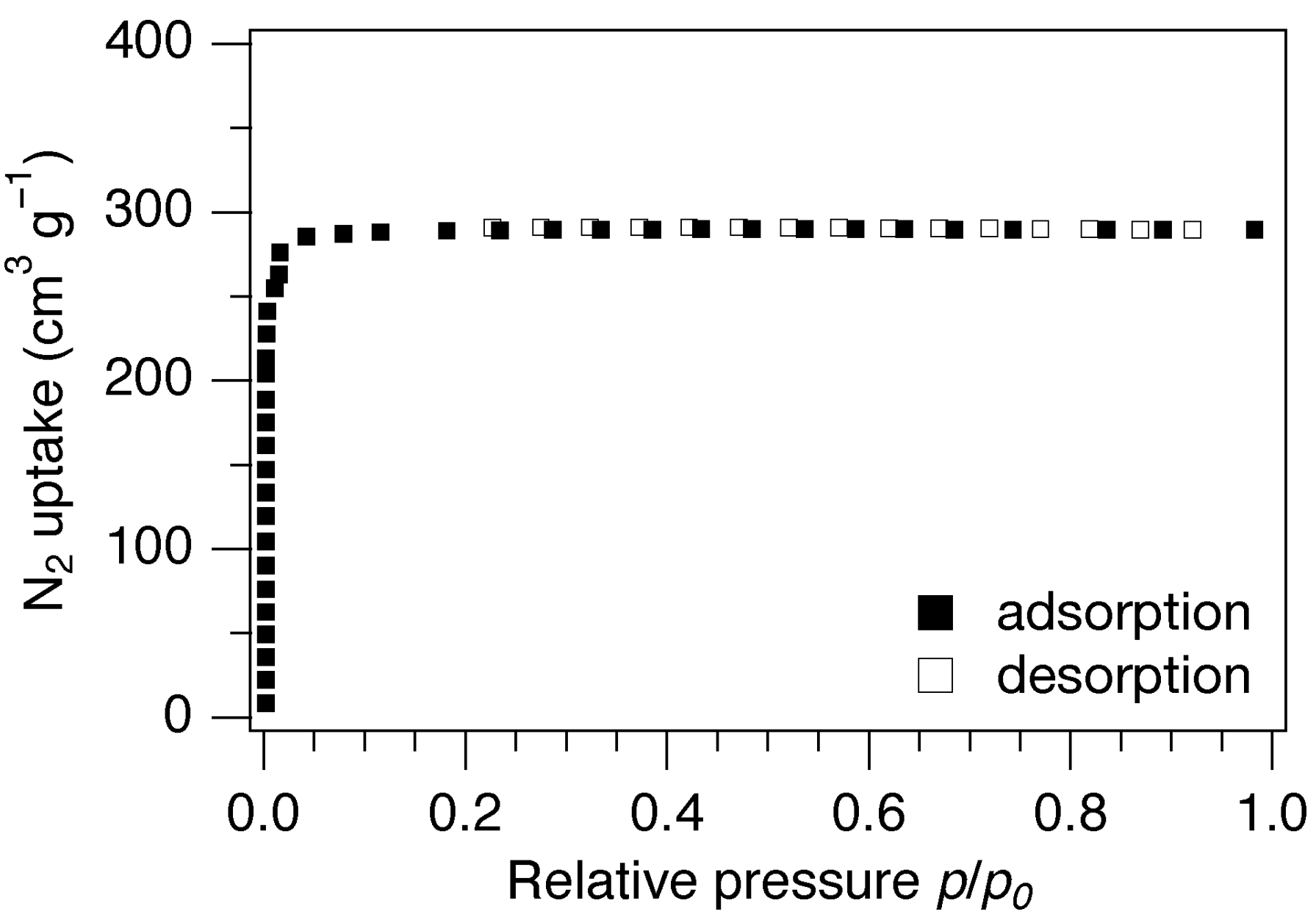

Fig. S4. N_2 gas sorption isotherms of **1** after activation, measured at 77 K. It shows a type I isotherm typical for a microporous material and there is no hysteresis between adsorption and desorption traces. The maximum N_2 uptake volume is consistent with the previously reported value.[S2]

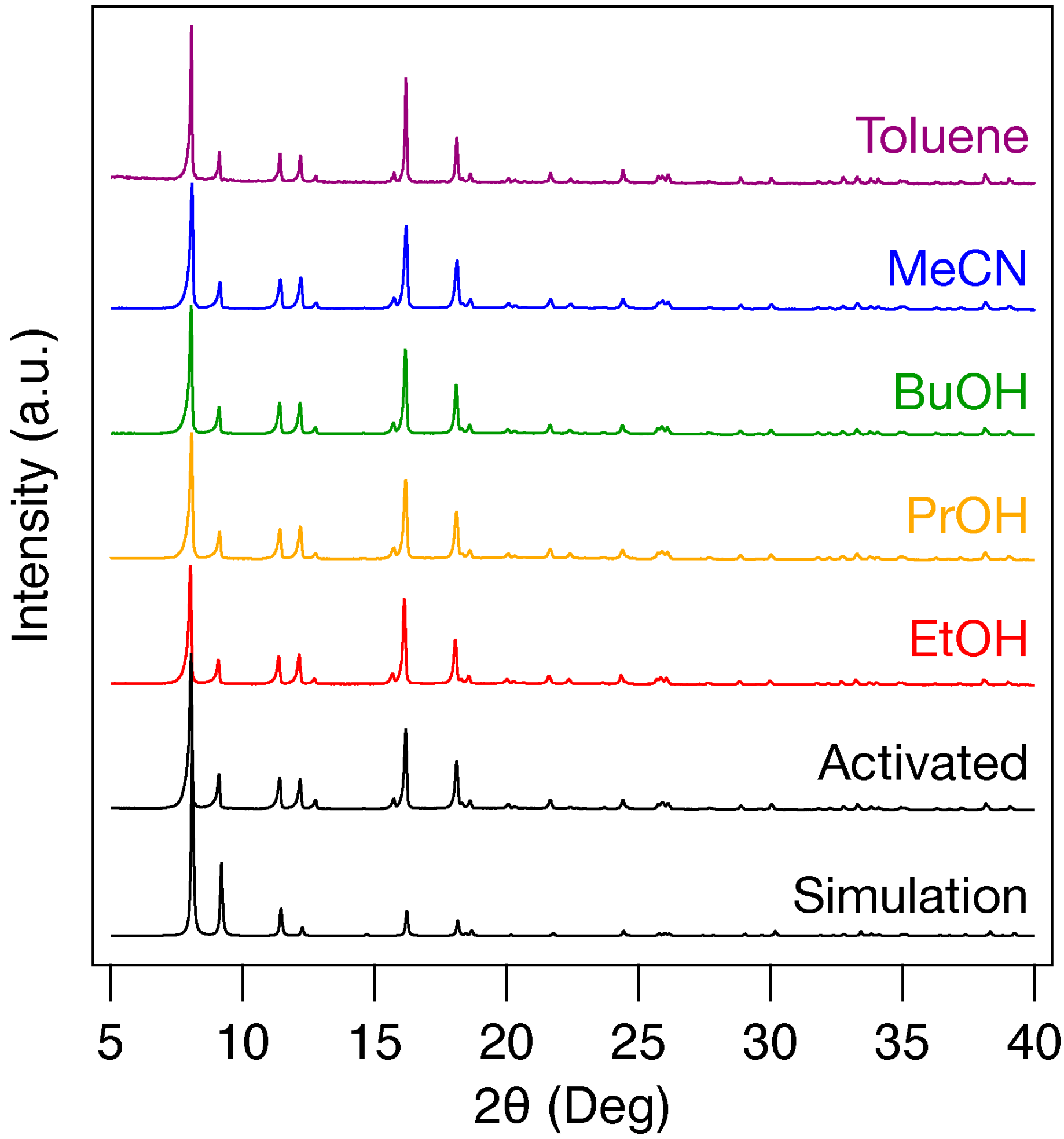

Fig. S5. PXRD data of **1**. The bottom black line represents the simulated PXRD pattern of **1**.[S2] The observed PXRD patterns for the activated **1** and those immersed in the given organic solvents for one week were all in good agreement with the simulated pattern, confirming the phase purity and high stability of **1** in the solvents used in this study.

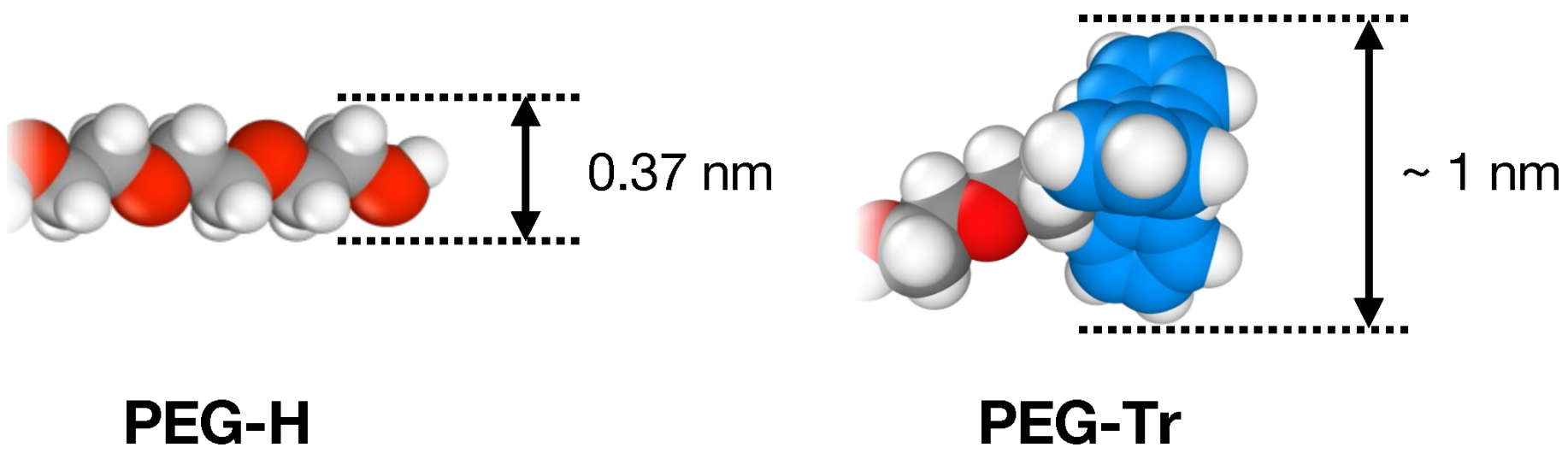

Fig. S6. Molecular mechanics models of the terminal structures of **PEG-H** (left) and **PEG-Tr** (right). The PEG chain thickness is approximately 0.37 nm, whereas the trityl terminus is approximately 1 nm and is larger than the 0.57 nm pore aperture of **1**. Atom colors: C, gray for PEG main chain/blue for aromatic rings; O, red; H, white.

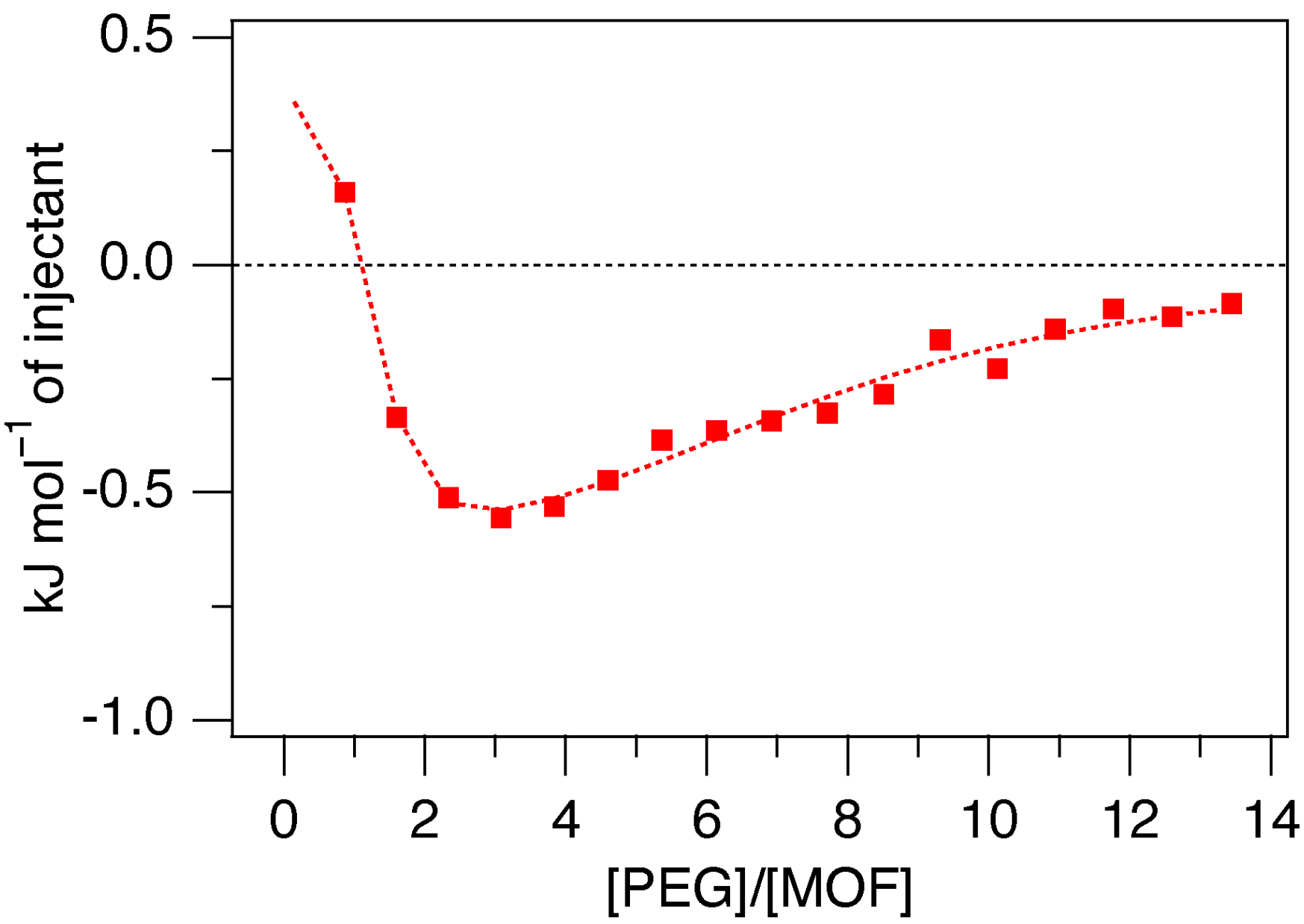

Fig. S7. Representative normalized injection heat profile for **PEG-H** adsorption into **1** at 313 K in EtOH, plotted as a function of [PEG]/[MOF]. The measurement parameters differ slightly from those used in Fig. 3, summarized in Table S6. M_0 = 4.2 mM, C_0 = 292 mM, Injection = 15 μL, and interval = 1.67 h. Dashed line represents the fit to the two-set model. The resulting parameters for the two distinct adsorption modes, Modes 1 and 2, are listed below. $N_1 = 0.94 \pm 0.26$, $K_1 = (10.4 \pm 22.5)\ 10^3\ \mathrm{M}^{-1}$, $\Delta H_1 = 0.50 \pm 0.62$ kJ mol^{-1}, $\Delta G_1 = -24.1 \pm 5.7$ kJ mol^{-1}, $T\Delta S_1 = 24.6 \pm 5.1$ kJ mol^{-1}, $N_2 = 6.35 \pm 1.02$, $K_2 = 150 \pm 69$ M^{-1}, $\Delta H_2 = -0.82 \pm 0.24$ kJ mol^{-1}, $\Delta G_2 = -13.0 \pm 1.2$ kJ mol^{-1}, $T\Delta S_2 = 12.2 \pm 1.4$ kJ mol^{-1}. Errors represent standard uncertainties from fitting (N, K, and ΔH) or error propagation (ΔG and $T\Delta S$).

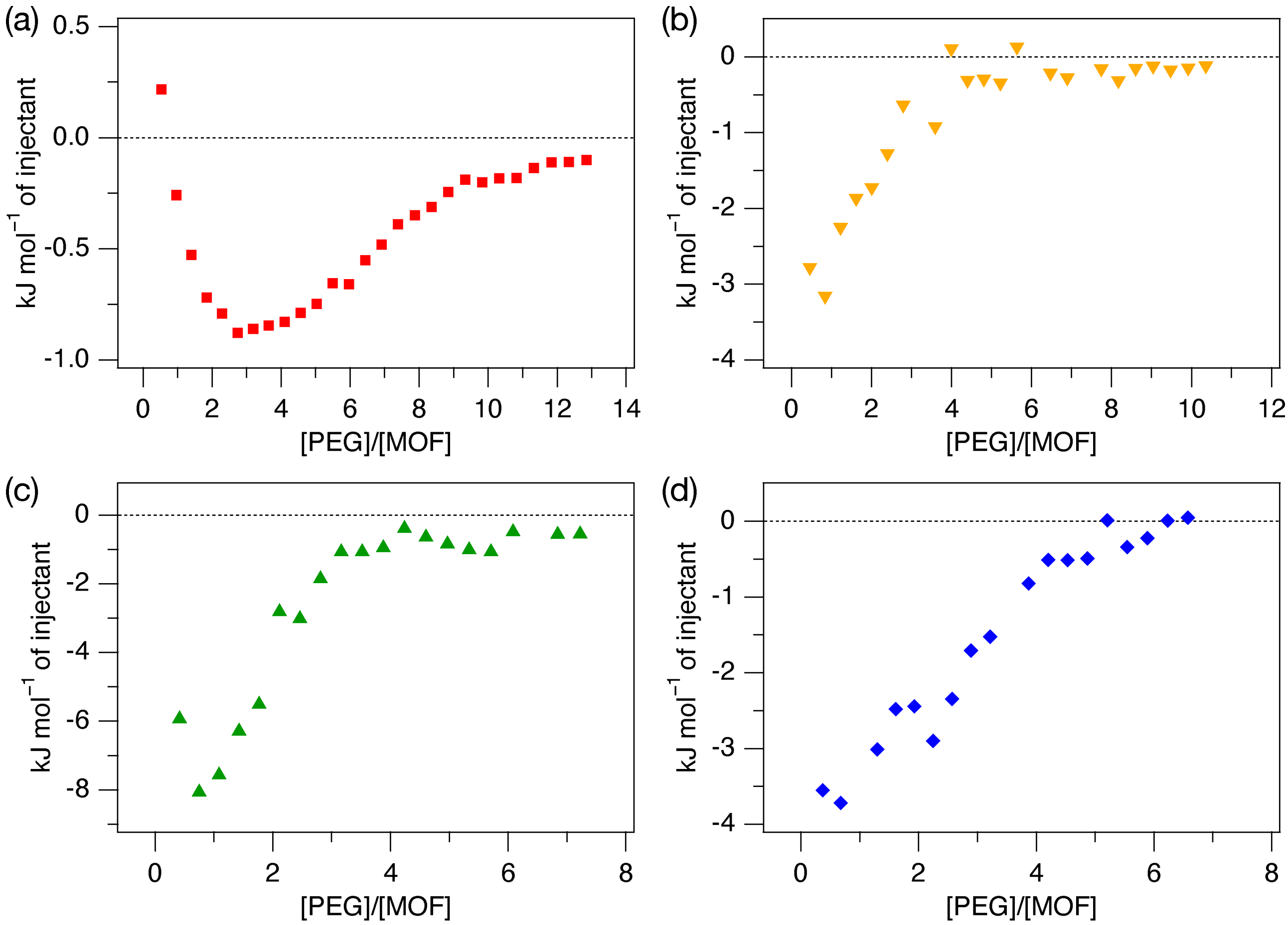

Fig. S8. Normalized injection heat profiles for **PEG-H** adsorption into **1** at 313 K measured in (a) EtOH, (b) PrOH, (c) BuOH, and (d) MeCN, plotted as a function of [PEG]/[MOF].

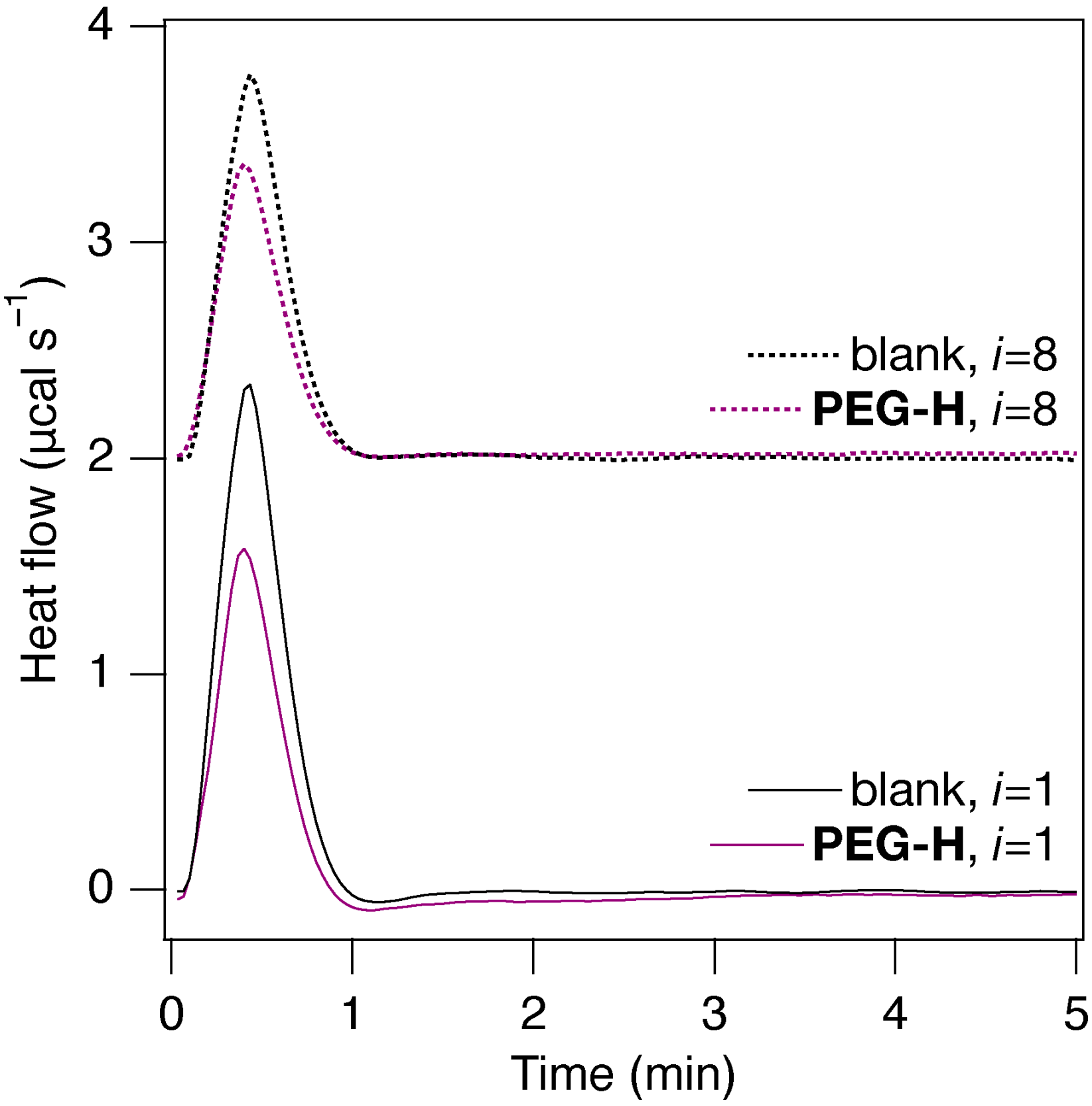

Fig. S9. Enlarged thermograms showing the first (below, solid lines) and eighth (above, dashed lines) injection peak for **PEG-H** experiments at 313 K in toluene. Black and purple traces correspond to the blank experiment and the suspension of **1**, respectively. In all cases, the heat flow returned to the baseline within a few minutes, indicating that only the immediate heat exchange associated with dilution (and possibly surface adsorption on the external surface of **1**) was observed.

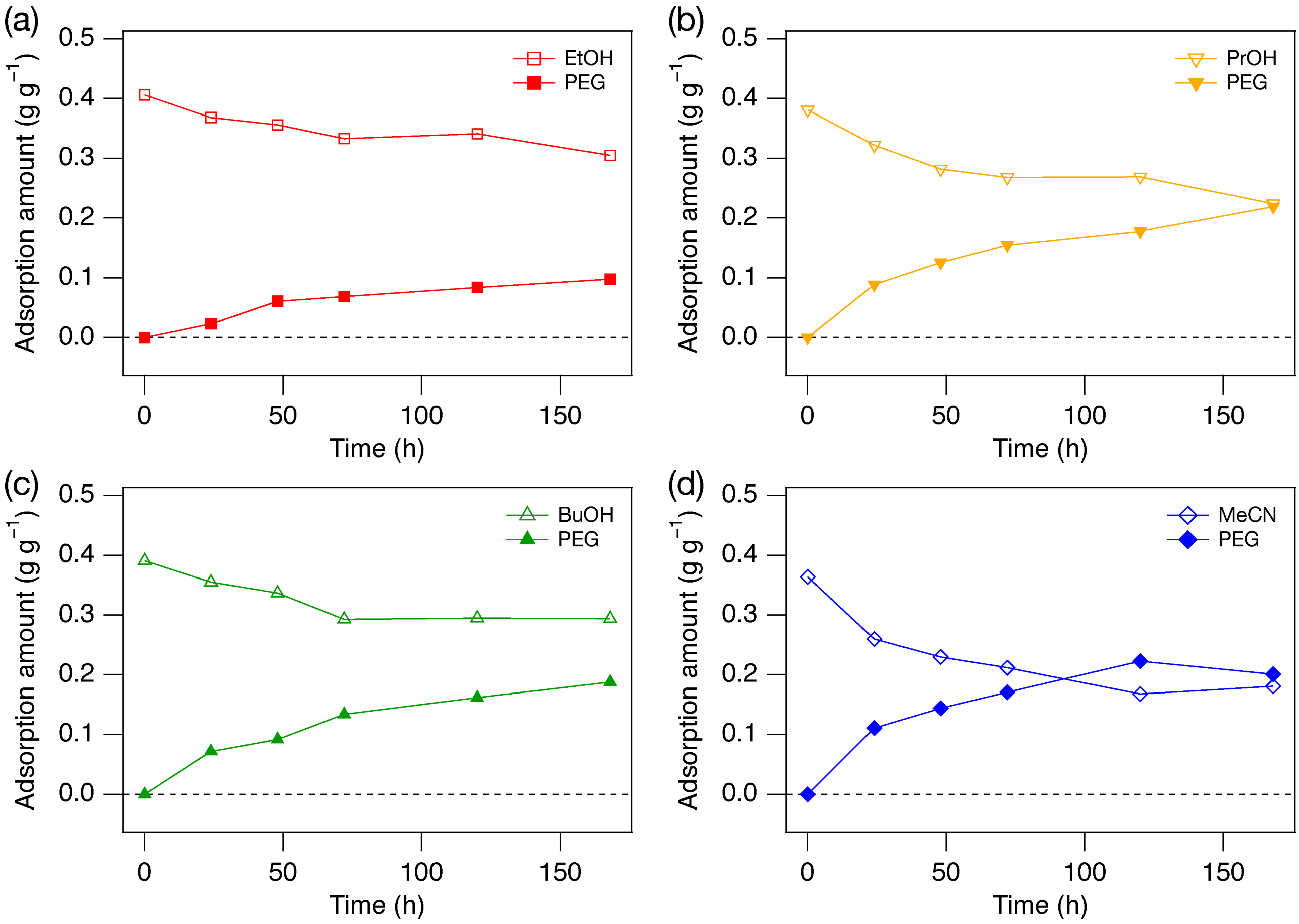

Fig. S10. Time-course changes in the adsorbed amount of solvent and **PEG-H** into **1** in (a) EtOH, (b) PrOH, (c) BuOH, and (d) MeCN, estimated from ^{1}H NMR. See Section 5 in this SI for details.

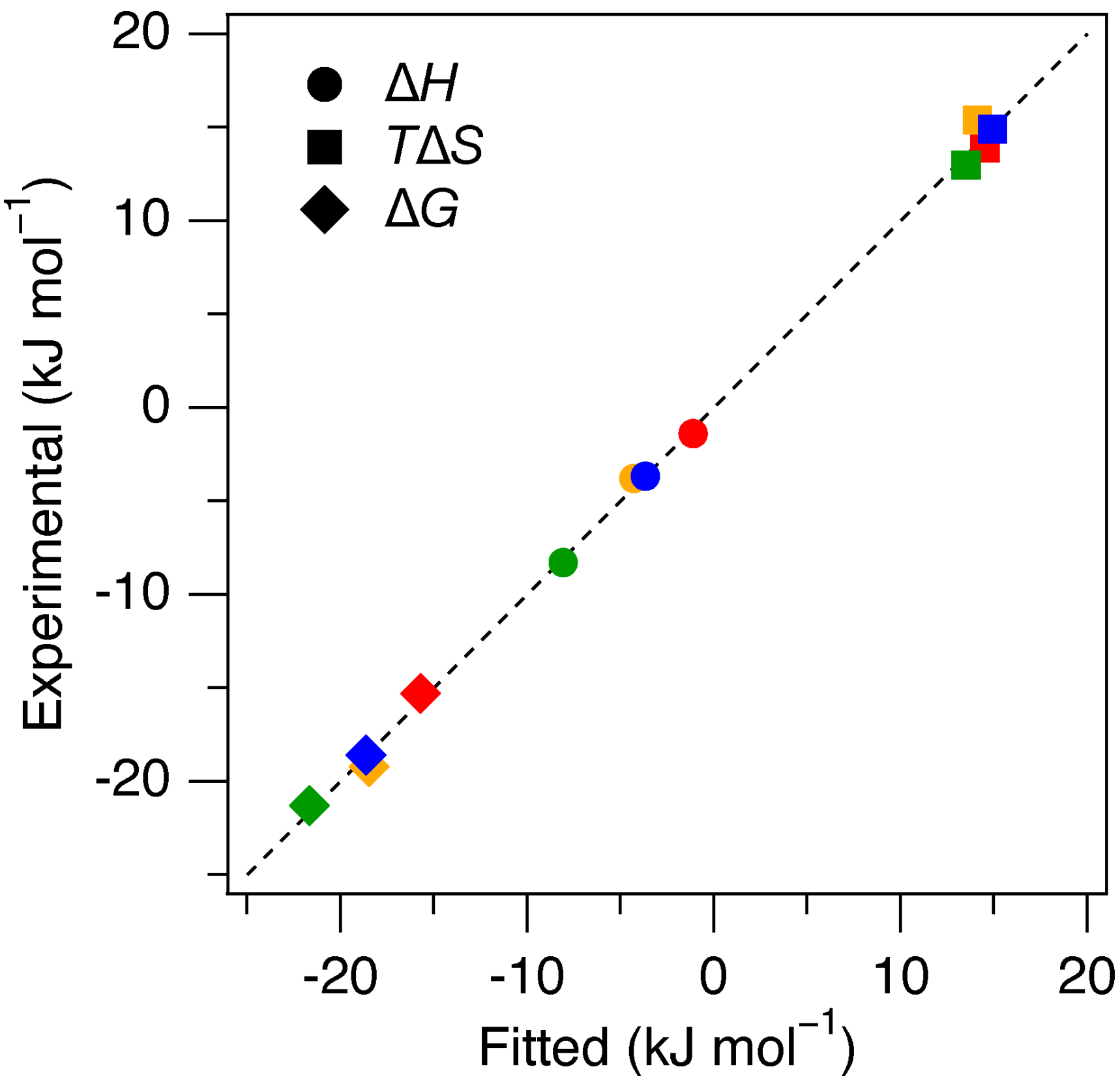

Fig. S11. Relationships between the experimental thermodynamic parameters (Table 2) and those calculated from Kamlet-Taft fitting analysis for the four solvents, EtOH (red), PrOH (orange), BuOH (green), and MeCN (blue) at 313 K. For EtOH, the parameters corresponding to Mode 2 adsorption from the two-set model fitting, which exhibited the dominant adsorption capacity, were used. The dashed line represents the identity line, $Y_{\mathrm{experimental}} = Y_{\mathrm{fitted}}$. Fitting procedure is described in Section 6 in this SI. The fitted coefficients are summarized in Table S5.

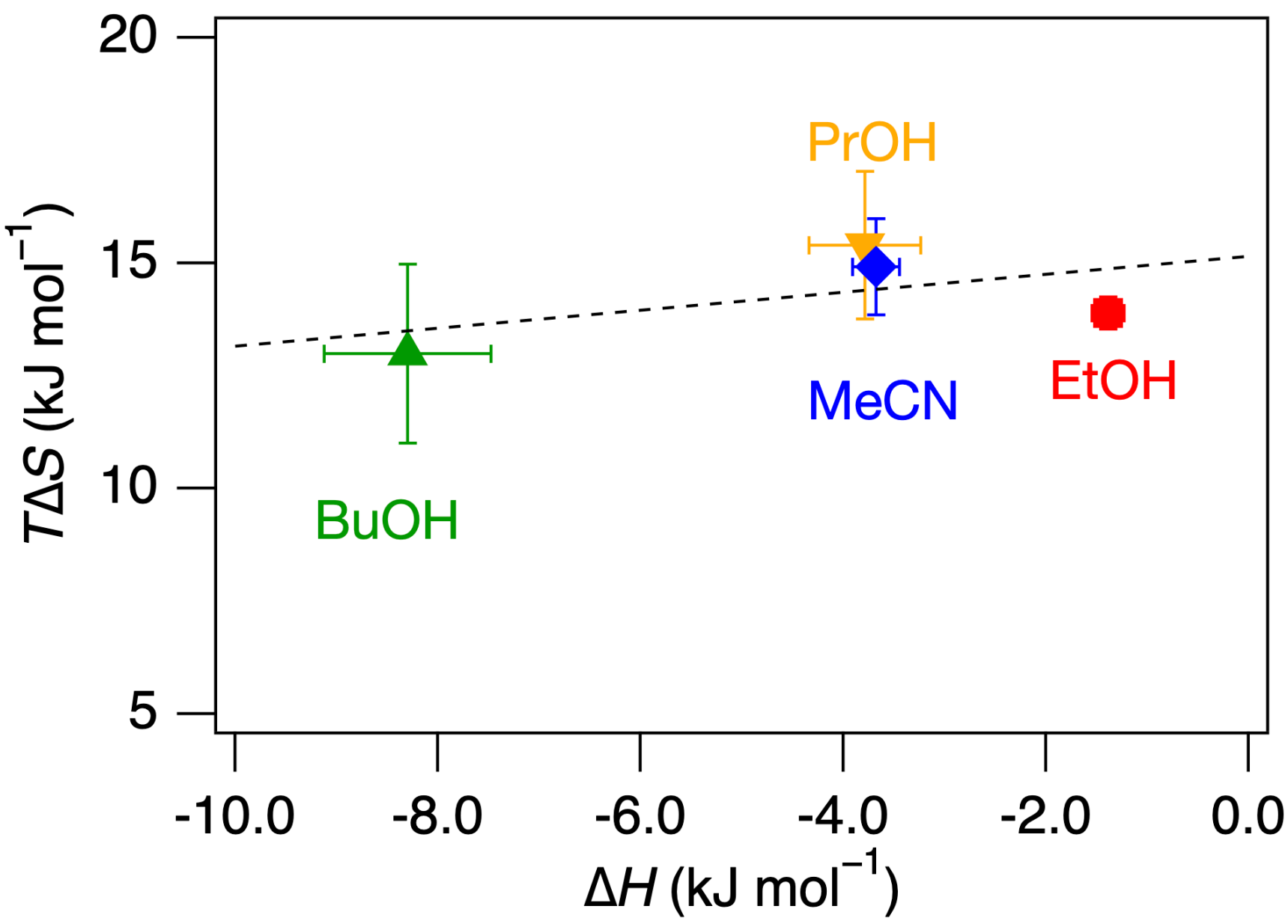

Fig. S12. Plot of $T\Delta S$ versus ΔH for **PEG-H** adsorption in the four different solvents. The parameters were obtained from the one-set model fitting for the given solvents except EtOH. For EtOH, the parameters corresponding to Mode 2 adsorption from the two-set model fitting, which exhibited the dominant adsorption capacity, were used. The dashed line represents a linear correlation with a slope of 0.20 ± 0.2 and an intercept of 15.1 ± 1.1 kJ mol^{-1}. Error bars represent standard uncertainties from fitting (ΔH) or error propagation (ΔG and $T\Delta S$).

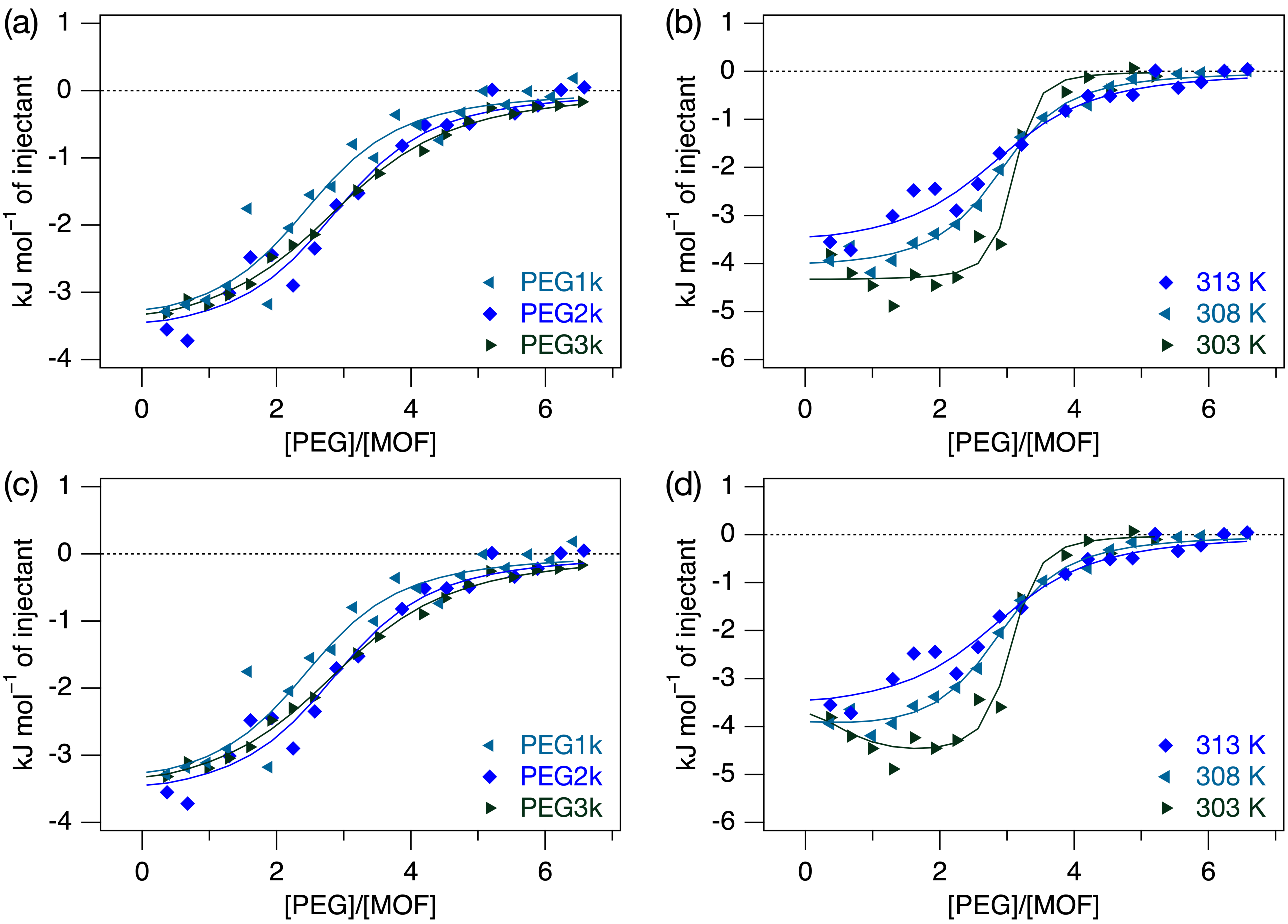

Fig. S13. Normalized injection heat profiles for PEG adsorption into **1** in MeCN, measured (a, c) with the three different molecular weights, 1, 2, and 3 kg mol^{-1} at 313 K, and (b, d) at the three different temperatures, 313 K, 308 K, and 303 K. Solid lines represent fits to (a, b) the one-set model, or to (c, d) the Fowler model. Fitted parameters from the Fowler model are summarized in Table S4.

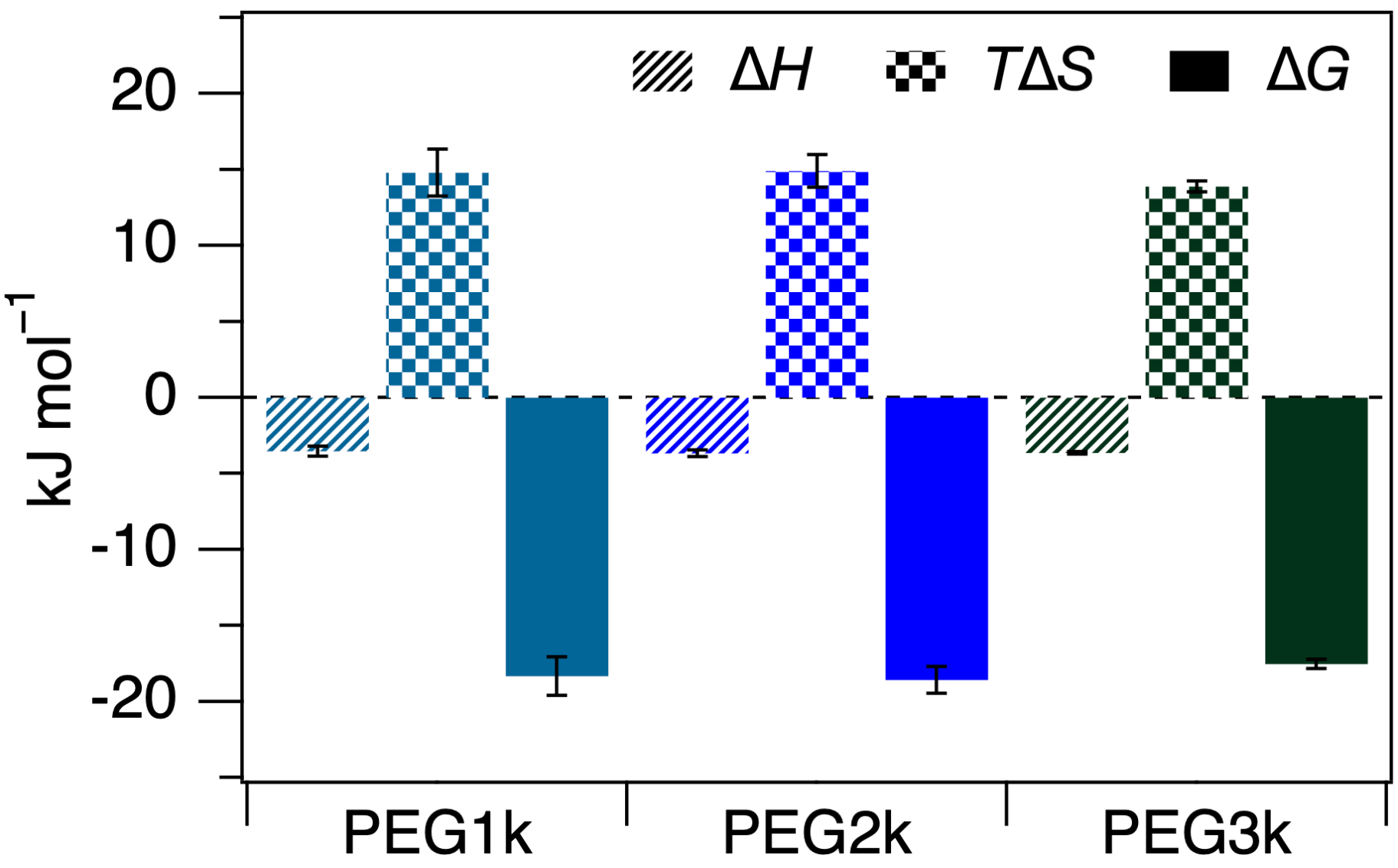

Fig. S14. Molecular-weight (MW) dependence of Δ*H*, *T*Δ*S*, and Δ*G* for PEG adsorption into **1** in MeCN, obtained from the one-set model fitting. Error bars represent standard uncertainties from fitting (Δ*H*) or error propagation (Δ*G* and *T*Δ*S*). The thermodynamic parameters per PEG repeating unit are almost identical within the studied MW region.

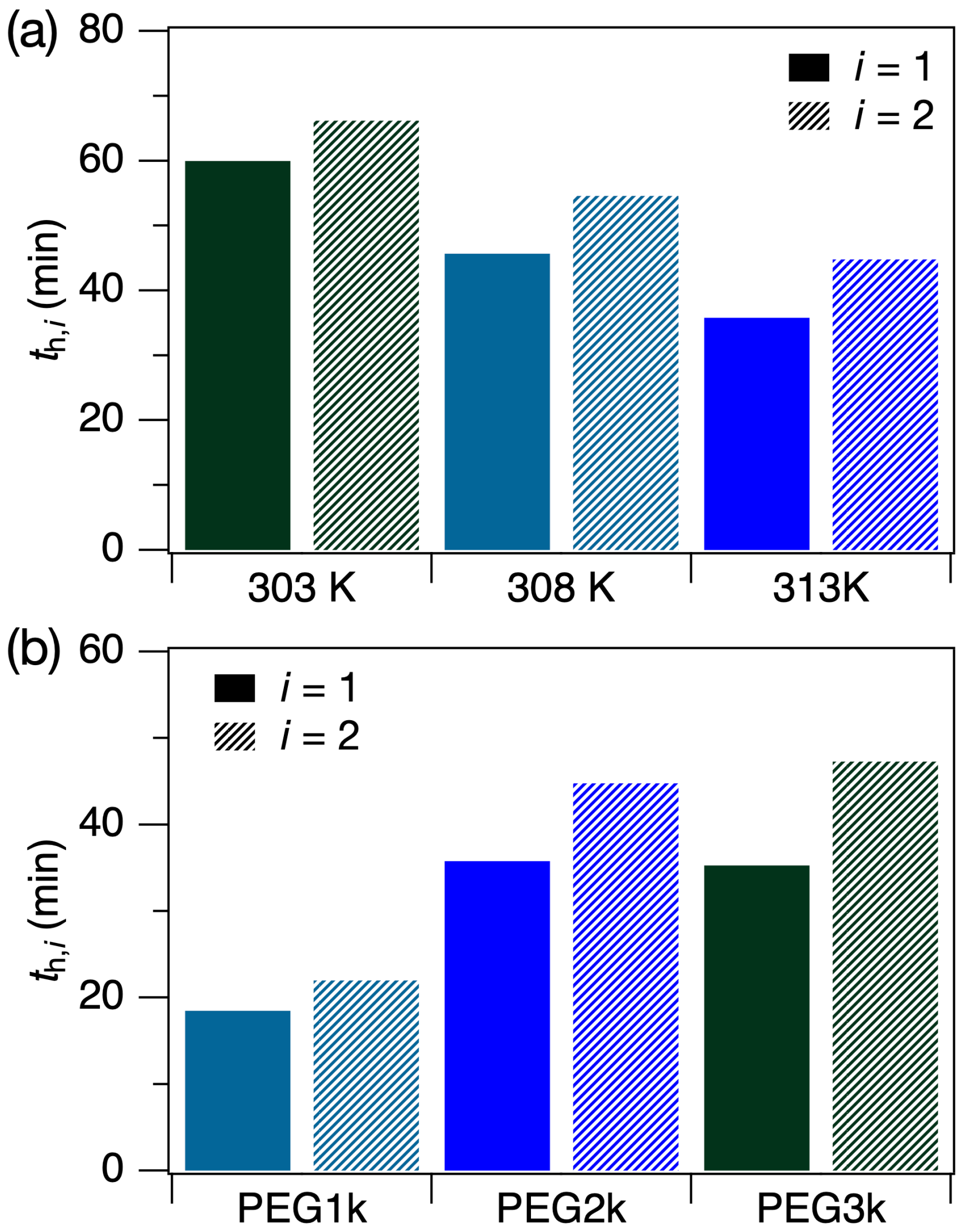

Fig. S15. (a) Temperature- and (b) molecular weight-dependence of the time required for the time-integrated heat to reach half of the total heat for first and second thermograms, $t_{h,1}$ (filled) and $t_{h,2}$ (hatched), for PEG adsorption into **1** in MeCN.

9. Supporting References

S1 B. Le Ouay, C. Watanabe, S. Mochizuki, M. Takayanagi, M. Nagaoka, T. Kitao and T. Uemura, *Nat. Commun.*, 2018, **9**, 3635.

S2 H. Chun, D. N. Dybtsev, H. Kim and K. Kim, *Chem. – Eur. J.*, 2005, **11**, 3521–3529.

S3 A. B. Greytak, S. L. Abiodun, J. M. Burrell, E. N. Cook, N. P. Jayaweera, M. M. Islam and A. E. Shaker, *Chem. Commun.*, 2022, **58**, 13037–13058.

S4 M. J. Kamlet, J. L. M. Abboud, M. H. Abraham and R. W. Taft, *J. Org. Chem.*, 1983, **48**, 2877–2887.

S5 N. Oe, N. Hosono and T. Uemura, *Chem. Sci.*, 2021, **12**, 12576–12586.

S6 M. W. Freyer and E. A. Lewis, *Methods Cell Biol.*, 2008, **84**, 79–113.
